# Integrated application of sugarcane by-product-derived organic fertilizer (SOFA) and mineral nitrogen enhances yield, fruit quality, and soil properties of eggplant (*Solanum melongena* L.) in sandy soil conditions

**DOI:** 10.1038/s41598-026-60547-1

**Published:** 2026-07-07

**Authors:** Abdel-Haleem A. H. El-Shaieny, Ahmed N. Gad El Rab, Hosny M. Farrag, Abeer Abd EL Moiez Ahmed Bakr, Safaa El-Nahas

**Affiliations:** 1 Horticulture Department, Faculty of Agriculture, Qena University, Qena, 83523 Egypt; 2 Research and Development Unit, Qus Sugarcane Factory (SCF), Qena, Egypt; 3 Soil and Water Department, Faculty of Agriculture, Qena University, Qena, 83523 Egypt; 4 Chemistry Department, Faculty of Science, Qena University, Qena, 83523 Egypt

**Keywords:** *Solanum melongena*, Sugarcane byproducts, Soil health, Integrated nutrient management, Sustainable agriculture, Anthocyanins, Environmental sciences, Plant sciences

## Abstract

Low soil fertility and restricted nutrient bioavailability are critical limiting factors for sustainable vegetable production, particularly in marginal sandy soils. This study addressed these challenges by investigating the efficacy of a novel sugarcane-derived molasses treated (SOFA) as a soil conditioner and nutrient source for eggplant (*Solanum melongena* L.), a crop for which comprehensive studies on such integrated approaches in these soil types are limited. Field experiments were conducted over two consecutive growing seasons to evaluate the impact of SOFA, applied either independently or in combination with mineral nitrogen (N), on soil physicochemical properties and crop performance.The study results demonstrated that integrated fertilization significantly improved soil physicochemical properties and crop performance. The treatment T3 (50% mineral *N* + 50% SOFA) achieved the highest total fruit yield (8.84 kg plant^−1^), representing a  111% increase compared to the control (4.18 kg plant^−1^). Similarly, T2 (75% *N* + 25% SOFA) significantly enhanced vegetative growth, chlorophyll content, and fruit nutritional quality. Soil organic matter increased by up to 56%, while available nitrogen, phosphorus, and potassium increased by up to 240%, 209%, and 67%, respectively, compared to the control. Moreover, SOFA application contributed to moderating soil alkalinity and improving nutrient availability, despite slight increases in electrical conductivity at higher application rates. Eggplant fruit quality traits were also significantly improved, with total soluble solids (TSS) increasing by  42% and anthocyanin content by  49% under optimized treatments. Economic analysis revealed that T2 and T3 achieved the highest profitability, with investment factors approaching 5.0, indicating superior economic efficiency. These findings highlight the potential of treated molasses-based amendments (SOFA) as a sustainable strategy for restoring marginal sandy soils and optimizing the productivity and nutritional quality of *S. melongena.*

**Supplementary Information:**

The online version contains supplementary material available at 10.1038/s41598-026-60547-1.

## Introduction

Low soil fertility, particularly in sandy soils, poses a significant challenge to sustainable agricultural productivity due to their poor water retention, low organic matter content, and rapid nutrient leaching^1–3^. While traditional organic amendments have long been recognized for their soil-improving properties, the intensification of modern agriculture has led to an increased reliance on synthetic chemical fertilizers^4^. However, this approach often overlooks the holistic benefits of organic matter in enhancing soil structure, microbial activity, and nutrient cycling, necessitating a more integrated nutrient management strategy.

Molasses, a significant byproduct of sugarcane and sugar beet processing, is a viscous, dark syrup rich in sugars (45–65%), nitrogen (0.43%), phosphorus (0.06–0.7%), and potassium (2.89–4%), alongside various micronutrients^5–9^. Historically, organic materials were central to agricultural practices; however, the intensification of agriculture has led to a reliance on chemical fertilizers^10^.

Egypt’s sugarcane industry, with roots dating back to 710 AD, is concentrated in Upper Egypt, producing approximately 16 million tonnes of cane annually across eight factories^11,12^. This process generates substantial quantities of byproducts, including 30% bagasse, 4% molasses, 3.5% filter mud/cake, and 0.4% furnace ash, amounting to millions of tons of residues annually^13^. The effective management and utilization of these residues present a critical opportunity for sustainable agriculture.

Previous research has demonstrated the beneficial effects of molasses application in agriculture. Studies have shown that molasses can enhance the uptake of micronutrients (Zn, Cu, Fe, Mn) in corn and wheat, improve nutrient uptake and yield in cabbage, and increase soil nitrogen and potassium while reducing available phosphorus^14–16^. Specifically, molasses application has been reported to improve plant growth, leaf area, and biomass in tomato plants under saline conditions^17^. Furthermore, sugarcane byproducts like molasses and vinasses have been shown to boost vegetative growth and fruit yield in sweet pepper, leading to higher quality produce and improved benefit-cost ratios by reducing reliance on mineral fertilizers^18,19^. Molasses have also been found to increase total nitrogen and potassium in soil, enhance growth traits (shoot length, leaf number, chlorophyll content) in peas^20,21^, and significantly improve root yield and quality in sugar beet, with soil applications proving more effective than foliar applications^22^. The combined application of molasses with organic fertilizers has been shown to substantially increase soil organic carbon, nitrogen, and potassium, while decreasing soil pH, ultimately leading to higher yields in spinach^23^. These findings underscore the potential of sugarcane industry byproducts to reduce fertilizer requirements, increase soil organic matter, and serve as components in integrated nutrient management strategies^24^.

Eggplant (*Solanum melongena* L.) is a main crop in Egypt, cultivated extensively during the summer season and valued for its rich nutritional content, including carbohydrates, protein, and essential vitamins and minerals^25,26^. Despite the clear benefits and the substantial availability of sugarcane residues in Egypt, there remains a mismanagement of these valuable resources. This research endeavors to address this gap by exploring achievable solutions through the recycling of sugarcane residues to produce organic nutrient sources.

Despite its significance, there is a notable research gap concerning the comprehensive evaluation of integrated organic-mineral fertilization strategies, particularly those utilizing sugarcane-derived amendments, for *S. melongena* cultivation in challenging sandy soil environments. Understanding the synergistic effects of such amendments is vital for developing sustainable fertilization practices.

In this context, our investigation aimed to evaluate a novel organic fertilizer, SOFA, derived from sugarcane industry byproducts, and its potential as a partial replacement for mineral fertilizers in S. melongena cultivation under field conditions. The study sought to identify optimal organic-mineral integration ratios to enhance soil fertility and crop productivity, thereby contributing to sustainable agricultural practices in regions characterized by poor soil quality soils.

## Materials and methods

Field experiment and plant material.

### Experimental site and soil characteristics

Field experiments were conducted during the 2023 and 2024 growing seasons at the Experimental Research Farm, Faculty of Agriculture, Qena University, Qena, Egypt (latitude 26° 11′ 22.2″ N, longitude 32° 44′ 25.5″ E; 81 m above sea level). Prior to experiment initiation, a composite soil sample (0–30 cm depth) was collected and analyzed for physicochemical properties^27^. The experimental site featured a sandy loam soil with the following characteristics: pH (1:2.5 soil: water suspension) 7.99, electrical conductivity (EC) 0.55 dS/m, calcium carbonate (CaCO3) 9.98%, organic matter 0.74%, total nitrogen (N) 0.015%, available phosphorus (P) 3.41 mg/kg, and available potassium (K) 114 mg/kg. Soil analyses were performed according to standard methods^28^.

### Plant material and cultural practices

Seeds of the commercial eggplant cultivar ‘Black Beauty’ were sown in a nursery under a plastic house on January 10th in both growing seasons. Seedlings were transplanted to the field in the first week of March. All agricultural practices followed the recommendations of the Egyptian Ministry of Agriculture. Each experimental plot measured 2.5 m in length and 4 m in width, resulting in a total area of 10 m^2^. Plots contained five ridges, each 50 cm apart, oriented north south. Harvesting commenced 90 days after transplanting and continued at 5-day intervals for 50 days.

### Fertilization program

The recommended mineral NPK fertilizers were applied as follows: 378 kg ha^−1^ ammonium nitrate (33.5% N) per hectare, 250 kg calcium superphosphate (15.5% P_2_O_5_) per hectare, and 150 kg potassium sulfate (48.52% K_2_O) per hectare. These fertilizers were applied in three split doses: 30% one month after transplanting, 35% two months after transplanting, and the remaining 35% three months after transplanting.

### Experimental design and treatments

The field experiment was arranged in a Randomized Complete Block Design (RCBD) with three replications. The experimental area was divided into three main blocks (28 m × 4 m each), separated by 1 m buffer zones. Each block comprised eight plots (2.5 m × 4 m, 10 m^2^ each), also separated by 1 m buffer zones. The study included five treatments, representing different combinations of SOFA and mineral fertilizers, based on the recommended nitrogen dose for sandy loam soil (378 kg N/ha):

T0: 100% recommended mineral nitrogen (378 kg N ha^−1^; control).

T1: 100% SOFA (nitrogen equivalent to 378 kg N ha^−1^).

T2: 75% mineral nitrogen (283.5 kg N ha^−1^) + 25% SOFA (94.5 kg N ha^−1^ equivalent).

T3: 50% mineral nitrogen (189 kg N ha^−1^) + 50% SOFA (189 kg N ha^−1^ equivalent).

T4: 25% mineral nitrogen (94.5 kg N ha^−1^) + 75% SOFA (283.5 kg N ha^−1^ equivalent).

The quantities of SOFA were calculated based on its total nitrogen content to match the equivalent N dose. For instance, 100% SOFA equivalency corresponded to 9600 L/ha (0.96 L/m^2^), 75% to 7100 L/ha (0.71 L/m^2^), 50% to 4800 L/ha (0.48 L/m^2^), and 25% to 2400 L/ha (0.24 L/m^2^). SOFA was applied through fertigation in three equal splits: 4 weeks after transplanting, 8 weeks after transplanting, and 12 weeks after transplanting.

### SOFA fertilizer preparation and characterization

SOFA (Sugarcane Organic Fertilizer Amendment) was prepared from a mixture of horticultural crop residues, tree leaves, field crop wastes, poultry litter, and cow dung collected from the Experimental Research Farm, Faculty of Agriculture, Qena University. These organic wastes were digested, air-dried, mechanically crushed, and then composted using the Passively Aerated Composting Technique (PACT). The composting piles were mechanically flipped and watered weekly for the first month, then bi-weekly thereafter, for a total of 100 days. A composite sample of the resulting compost was analyzed. Molasses treated SOFA was prepared by mixing the compost with tap water at a 1:10 (w/v) ratio, agitated manually for 7 days, and then purified to remove solid particles. The detailed physicochemical properties and nutrient composition of the molasses treated SOFA are presented in Table 1.

**Table 1:** Physicochemical properties and nutrient composition of SOFA.

Parameter	Value	Unit
Total nitrogen (N)	3.94	%
Total phosphorus (P)	2.78	%
Total potassium (K)	7.08	%
Organic carbon (C)	34.87	%
Organic matter (OM)	45.76	%
pH	7.96	–
EC	14.6	dS/m
Micronutrients		
Fe	149.5	mg/kg
Mn	21.00	mg/kg
Zn	11.75	mg/kg
Cu	8.40	mg/kg

#### SOFA product composition

The novel sugarcane-derived organic fertilizer amendment (molasses treated) (SOFA) is formulated from three primary components:

1. Component A: A liquid byproduct stream obtained directly from the sugarcane manufacturing process.
2. Component B: Synthesized nano-hydroxyapatite (nHAp), serving as a controlled-release phosphorus source and soil conditioner.
3. Component C: A proprietary blend of essential plant micronutrients.

#### Fabrication of nano-hydroxyapatite (nHAp)

**Fig. 1:**
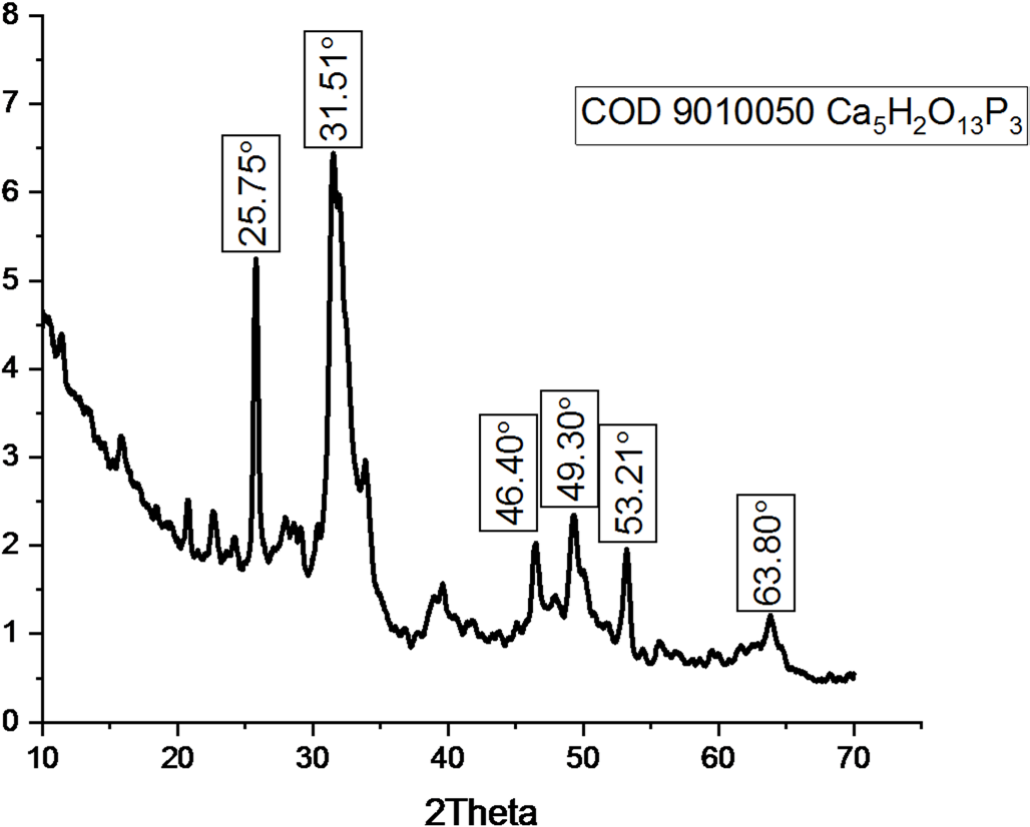
XRD pattern for hydroxyapatite samples.

The nano-hydroxyapatite (nHAp) utilized in SOFA was synthesized via a wet chemical precipitation method, as schematically represented in Fig. 1. The detailed procedure is as follows: Initially, 10 g of a calcium salt precursor was dissolved in 100 mL of distilled water under continuous magnetic stirring until complete dissolution. The resulting solution was subsequently filtered to ensure the removal of any insoluble impurities. Following this, 20.5 mL of concentrated phosphoric acid (H3PO4), serving as the phosphorus source, was slowly added to the calcium solution over a period of 1 h, maintaining constant stirring. After achieving a homogeneous mixture, precipitation was induced by the dropwise addition of concentrated ammonium hydroxide (35%) until the solution pH reached 12, indicated by the formation of a distinct white precipitate. The resultant gel was then subjected to microwave irradiation at 170 W for 5 min to promote crystallization. The synthesized nHAp was subsequently aged at 40 °C for 20 h. Finally, the nHAp precipitate was isolated by filtration, thoroughly rinsed with deionized water to remove residual ions, and dried overnight at 60 °C.

#### Characterization of nano-hydroxyapatite

The synthesized nano-hydroxyapatite (nHAp) was comprehensively characterized using a suite of analytical techniques to confirm its physicochemical properties and nanoscale morphology. Fourier Transform Infrared (FTIR) spectroscopy was performed using a Nicolet Magna-FTIR-560 (USA) with the KBr pellet technique to identify functional groups and confirm the presence of phosphate and hydroxyl bands characteristic of hydroxyapatite. Powder X-ray Diffraction (XRD) analysis was conducted on a Brucker Axs-D8 Advance Diffractometer (Belgium) at ambient temperature, scanning in the 2θ range of 10°–70°, to determine the crystalline phase, purity, and crystallite size of the nHAp. Thermal properties were assessed using Thermogravimetric (TG) and Differential Scanning Calorimetry (DSC) curves, recorded with an automatically recording Shimadzu thermal analyzer (Model 50 H, Japan). The morphology and elemental composition of the synthesized nHAp were investigated using Scanning Electron Microscopy (SEM) coupled with Energy Dispersive X-ray (EDX) spectroscopy (Model FEI INSPECT S50), operating at 20 kV. Furthermore, the specific surface area, total pore volume, and average pore radius were determined by the Brunauer-Emmett-Teller (BET) method using an Automatic ASAP 2010 Micrometrics sorptometer (USA) at liquid nitrogen temperature (77.350 K).

#### SOFA product preparation

The final SOFA product was prepared through a standardized, multi-step mixing process:

1. Dilution of Component A: The liquid byproduct (Component A) from the sugar company was introduced into a stirred tank and diluted with distilled water. Continuous stirring was maintained at 1000 rpm to ensure homogeneity.
2. Incorporation of Component B: The prepared nano-hydroxyapatite (Component B) was added to the diluted Component A at a concentration of 20% (w/w).
3. Addition of Component C: The micronutrient blend (Component C) was subsequently incorporated at a concentration of 5% (w/w).
4. Homogenization: The mixture was continuously stirred for a minimum of 1 h to ensure thorough dispersion and complete integration of all components.
5. Concentration Adjustment: The total solid concentration of the final product was precisely adjusted to a Brix value (°Bx) of 75 using distilled water, as measured by a refractometer, to ensure consistent product quality.
6. Quality Control Sampling: A 500 mL sample was collected from each batch for comprehensive quality control analysis, including the determination of potassium, boron, nitrogen, phosphorus, organic matter, trace elements, and natural chelates.
7. Packaging: The final liquid product was packaged into bottles, while solid formulations were stored in paper bags, as appropriate for the intended application.

### Soil and plant measurements

#### Soil sample collection and analysis

Original soil samples (0–30 cm cultivated layer) were collected using a stainless-steel auger^29^. Total calcium carbonate (CaCO_3_%) was determined using a Collins calcimeter^30^. Soil organic carbon content was estimated by the modified Walkley and Black method^31^. Soil pH (1:2.5 soil: water suspension) was measured with a digital pH meter. Electrical conductivity (EC) (1:5 soil: water extract) was determined using an electrical conductivity meter^30^. Available phosphorus was extracted with 0.5 M NaHCO_3_ (pH 8.5)^32^ and quantified spectrophotometrically^30^. Available potassium was extracted with 1 N ammonium acetate (pH 7.0) and determined by flame photometry^30^. Total soil nitrogen was measured using the micro-Kjeldahl method^30^.

#### Eggplant growth, yield, and quality parameters

At 70 days after transplanting, five randomly selected plants per plot were assessed for vegetative growth and nutritional status. Plant height (cm) was measured at 16, 32, and 60 days after transplanting. Other parameters included number of branches, number of leaves per plant, and fresh and dry weights of leaves (g). Chlorophyll content was measured in mature leaves using a Konica Minolta Chlorophyll Meter (SPAD 502 plus, Japan).

At harvest (90 days after transplanting), total fruit yield per hectare was calculated from plot yield. Average fruit weight (g) and number of fruits per plant were also recorded. Fruit quality parameters were assessed from ten fruits per replicate: fruit diameter (cm) was measured with a digital caliper at the widest part, and fruit length (cm) was recorded. Fruit dry matter percentage was determined by drying samples at 105 °C for 4 h, followed by 70 °C until constant weight, calculated as (sample dry weight/sample fresh weight) × 100. Total soluble solids (TSS) were measured immediately using a digital refractometer. Anthocyanin content was estimated spectrophotometrically^33^ after extracting fruit peel samples with 10 mL of 1% HCl (w/v) in methanol overnight at 4 °C. Extracts were filtered, and absorbance was measured at 530 nm.

#### Plant nutrient uptake

Fresh fruits collected at harvest were air-dried and weighed. Plant tissue samples were oven-dried at 70 °C for 72 h. A 0.5 g aliquot of dried plant material was digested using a 20:5 mixture of sulfuric acid and hydrogen peroxide. Total nitrogen (N) content was determined by the Kjeldahl digestion method. Phosphorus (P) content was measured spectrophotometrically, and potassium (K) concentration was determined by flame photometry^30^.

### Economic study

To perform an economic analysis, the total cost of production and total income were calculated. To estimate the total outlay, the cost of all agricultural inputs and practices was used at prevailing market rates. The total income was calculated by multiplying the total fruit production by the yield unit price in the local market. Net returns were calculated by subtracting the total cost from the total income. The benefit-cost ratio was calculated according to Boardman et al.^34^. by dividing the gross return on total variable cost (Egyptian pound /ha).

### Statistical analysis

All collected data for plant and soil parameters were subjected to statistical analysis using a one-way analysis of variance (ANOVA), SAS 9.4 (SAS Institute, Inc., 2013) software^35^ for a Randomized Complete Block Design with three replicates across both growing seasons. Significant differences between treatment means were determined using the Least Significant Difference (LSD) method at a probability level of *P* < 0.05^36^.

The assumptions of analysis of variance (ANOVA) were verified prior to statistical analysis using the complete dataset from both growing seasons before pooling the data. The Shapiro–Wilk test indicated that the residuals for all measured variables followed a normal distribution (*P* > 0.05), while Levene’s test confirmed the homogeneity of variances among treatments (*P* > 0.05). (Supplementary Table S2). Therefore, the use of parametric ANOVA was considered appropriate. As the year × treatment interaction was not statistically significant for the majority of the studied traits, data from the two seasons were pooled and analyzed using the combined means of both years to enhance statistical robustness and minimize environmental variability between seasons.

## Results

### Composition of product (SOFA)

#### XRD analysis

The XRD pattern obtained for hydroxyapatite matched the standard card (COD 9010050) for the chemical structure of pure hydroxyapatite samples and the prominent peak positions corresponding to the planes at angles 2Ө= (25.80, 31.71,49.40, and 53.10). The hydroxyapatite’s crystallite size was 22.12 nm, which was in the nano-size range^37^ as displayed in Fig. 1.

#### SEM analysis

**Fig. 2:**
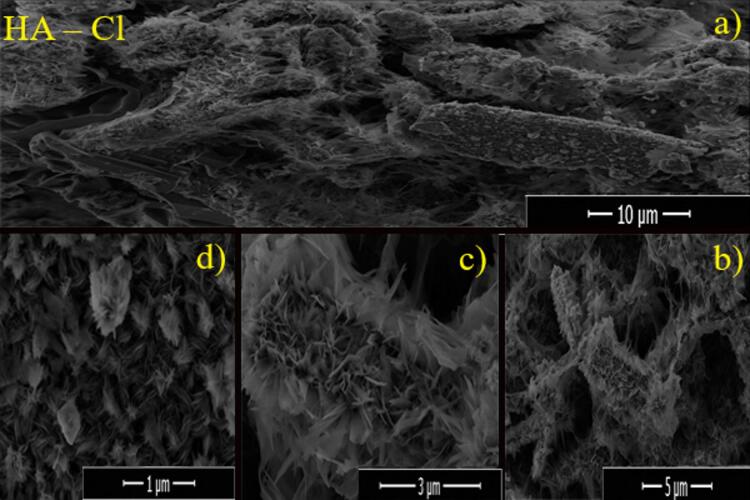
The SEM photos of prepared hydroxyapatite.

Scanning Electron Microscopy (SEM) was utilized to show the morphology of the prepared hydroxyapatite material. Figure 2 shows SEM images of hydroxyapatite samples that appeared in a fibrous Coral reefs-like structure stacked together^37^. EDX analysis used to validate the elemental composition of hydroxyapatite samples. The synthetized sample had a Ca/P ratio of 1.67.

#### FTIR analysis

**Fig. 3:**
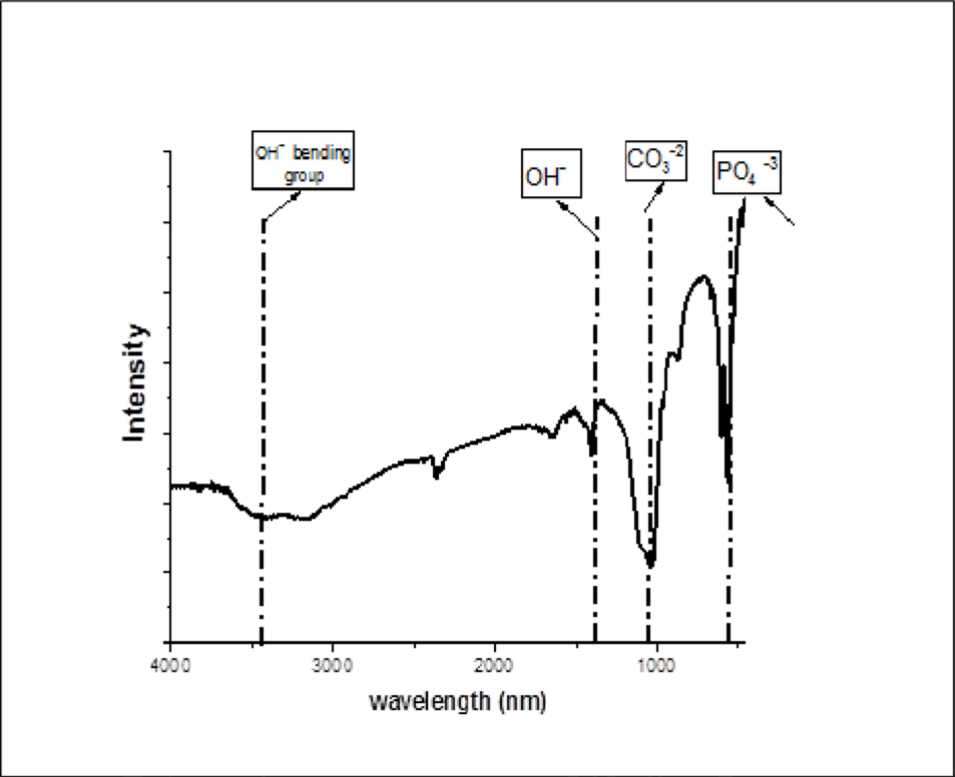
The FTIR spectrum for tested hydroxyapatite.

The functional groups of hydroxyapatite sample were verified using photospectroscopy analysis. The FT-IR spectrum of the synthesized nano-apatite was demonstrated in Fig. 3. The peak appears around 3600–3400 cm^−1^at 3455 cm^−1^ was attributed to the stretching OــــH while, around 1600–1500 cm^−1^ is attributed to the Oــــ H bending of the surface adsorbed water.

The distinctive bands of the phosphate for specific group of apatite were at 1061–1007 cm^−1^, 574–533 cm^−1^ at and 634–607 cm^−1^ were known as hydroxyapatite bands^38^. The PO_4_^-3^ group’s characteristic bands for the prepared HA sample were 1021 cm^−1^, 560 cm^−1^, and 610 cm^−1^. The carbonate (CO_3_^2−^) functional group appeared at wavenumbers 1405.91 cm^-1^ which was indicated by the vibration C–O band of (CO_3_^2−^) group^39^.

#### Thermogravimetric analysis

**Fig. 4:**
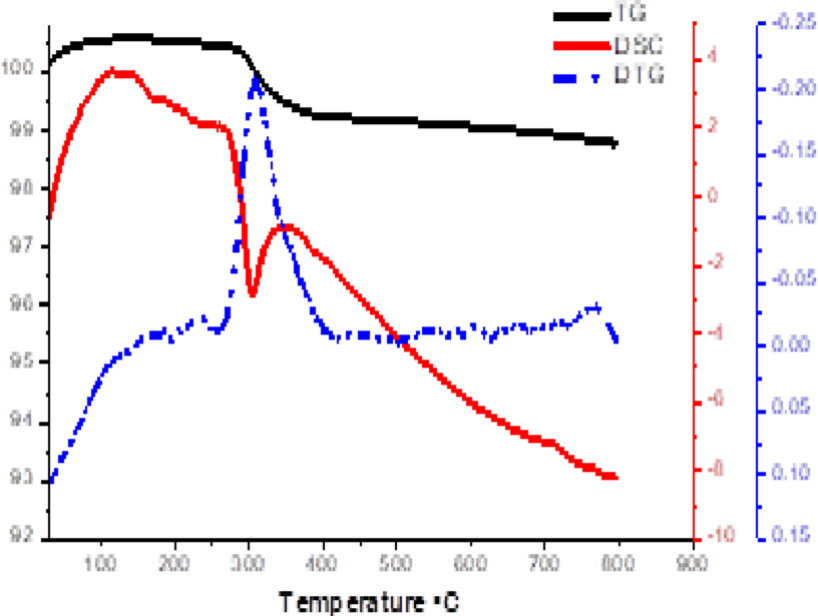
The Thermogravimetric for tested hydroxyapatite

Thermal analysis (TA) is a group of processes that monitor changes in a sample’s chemical structure over time as it is subjected to a temperature programmer. Mass, time and temperature of samples are tracked and recorded using a TGA diagram. Figure 4 depicts the TGA, DTG, and DSC curves for hydroxyapatite sample.

TG curves showed that the heating diagram was in one stage. The total weight loss for tested sample was about 1%, indicating that the sample was very thermally stable. The sample weight loss occurred at temperature below 300 °C. The decomposition of nitrates, ammonia, or intermediate components of gels to thermal decomposition products occurs at temperatures below 300 °C. The decomposition of nitrates or ammonia in the ingredients might be the cause of this loss^40^.

The information obtained by DTG curve displayed a single DTG peak at 308 °C. which has been recorded by DSC curves that revealed endothermic peak at 304 °C. owing to the decomposition of nitrates, ammonia, or intermediate components of gels^41^.

#### Surface area measurements

The BET technique is a well-known and commonly used method for estimating the specific surface area of solid materials, particularly those with open porosity. The hydroxyapatite sample showed high specific surface area (162.73 m^2^/g) as displayed in Table 2. The synthetic HA had typical I-type adsorption isotherms, indicating the presence of microporous materials with average pore radius 8.87 A°^37^.

**Table 2:** Surface data for synthesized hydroxyapatite samples.

Sample name	BET square (m^2^ g^−1^)	Total pore volume (cm^2^ g^−1^)	Aaverage pore radius (A°)
HA	162.73	0.07220	8.87

#### GC-Mass area analysis

Important components found in molasses include non-sugar organic matter (betaine and other amino acids, minerals and vitamins, and so forth) and carbs (glucose, sucrose, and fructose). Molasses can benefit from the addition of amino acids. It is evident that it includes certain organic acids, amino acids, and mono- and disaccharides. Additionally, phenolic acids such as ferulic acid, vanillic acid, syringic acid, p-hydroxybenzoic acid, and p-hydroxybenzaldehyde were found. Additionally identified are iso-orientin-7–3′-*O*-dimethylether and dehydrodiconiferyl alcohol-9′-*O*-D-gluopyranoside. Antioxidants and flavonoid derivatives were found in higher concentrations. The molasses sample has been found to include formic acid, acetic acid, aconitic acid, lactic acid, and trace amounts of malic, citric propionic, and n-butyric acids. 5-Hydroxymethylfurfural, oleic acid, acetic acid-phenyl-methylester, guanosine, 2-pentanone, 4-hydroxy-4-methyl, 4 H-pyran-4-one, 2,3-dihydro-3,5-dihydroxy-6-methyl, 9-hexadecenoic acid, and benzene are among its constituents^38^.

The supplementary Table S1 and Fig. S1 includes the total compounds analysis in SOFA, the results of the GC–MS analysis revealed a diverse profile of volatile and semi-volatile compounds detected at retention times (RT) ranging from 4.04 to 5.59 min. The identified compounds belong mainly to oxygenated derivatives, nitrogen-containing compounds, and aromatic hydrocarbons. The predominant compound across the chromatographic profile was 2-pentanone, 4-hydroxy-4-methyl- (C_6_H_12_O_2_; MW 116), which appeared at RT 4.24 and 4.34 min with the highest relative peak areas (3.35–5.78%), indicating it as the major constituent of the sample. Compounds detected at RT 4.56 min exhibited moderate abundance (4.89%), including 2-nitrohept-2-en-1-ol (C_7_H_13_NO_3_), clasto-lactacystin δ-lactone (C_10_H_15_NO_4_), and related nitrogenous and oxygenated structures, suggesting the presence of bioactive or oxidized metabolites. Aromatic hydrocarbons such as 1-ethyl-3-methylbenzene, 1-ethyl-4-methylbenzene, and 1,3,5-trimethylbenzene were detected at RT 4.94–5.59 min with relative areas of 2.44–4.48%, indicating a contribution of substituted benzene derivatives to the volatile fraction. Match factor (MF) values ranging from 624 to 936 and identification across multiple spectral libraries (Wiley Registry, Mainlib, NIST) support the reliability of compound assignment. Overall, the chromatographic profile demonstrates a predominance of oxygenated ketones and aromatic hydrocarbons, which may contribute significantly to the chemical and functional characteristics of the analyzed sample.

### Effects of partial replacement of N mineral fertilizer with SOFA on the vegetative growth of *S. melongena*

The results showed that positive significant changes in vegetative growth are characteristic of eggplant during both seasons as compared with the SOFA fertilizer and control treatment, these changes are as follows:

#### Plant height at 16, 32, and 60 days after transplanting

The effects of different fertilizer treatments had a certain effect on the plant height of eggplant, the results of *S. melongena* height data at 16, 32, and 60 days after transplanting are shown in Table 3. In the first 60 days, the eggplant height increased rapidly with different fertilizers treatments. The average plant height of treatment with T2 (75% RD *N* + 25% SOFA) was slightly higher than the other treatments after 16 DAP, which recorded 18.51 cm, which increased by 1.02 cm compared with control treatment. While the treatments T2, T3, and T4 was higher than the control treatment at 32 DAPS, which reached 36.81, 34.95, and 34.08 cm, respectively, which increased by 11.92, 6.26, and 3.62% compared to T0 control treatment. The results of eggplant height of all treatments were higher than the control treatment RD of N, the tallest plants were recoded with treatment T2 after 60 DAT, which increased by 9.6 cm compared with control treatment T0.

**Table 3:** Effects of fertilization treatments on plant height cm^−1^ 16, 32 and 60 ADP, (PH), in the cultivation of *Solanum melongena* in both seasons.

Treatments	2023			2024		
	PH_16_	PH_32_	PH_60_	PH_16_	PH_32_	PH_60_
T0	17.60 ± 0.22	32.78 ± 0.39	58.28 ± 0.36	17.37 ± 0.22	33.00 ± 0.00	58.00 ± 0.00
T1	16.76 ± 0.14	30.98 ± 0.64	66.14 ± 1.60	17.62 ± 0.14	34.32 ± 0.04	60.00 ± 0.00
T2	18.58 ± 0.30	36.62 ± 0.32	68.66 ± 0.34	18.44 ± 0.23	37.00 ± 0.00	69.33 ± 0.00
T3	17.37 ± 0.25	34.90 ± 0.45	60.08 ± 0.12	17.01 ± 0.25	35.00 ± 0.00	65.00 ± 0.00
T4	17.30 ± 0.23	34.17 ± 0.30	62.72 ± 1.19	17.16 ± 0.23	34.00 ± 0.00	62.00 ± 0.00
Significant	**	**	**	**	**	**
LSD 05	0.73	1.38	2.89	0.73	0.52	0.47

T0: Control; T1: 100% SOFA; T2: 75% RD + 25% SOFA; T3: 50% RD + 50% SOFA; T4: 25% RD + 75% SOFA. * and **, Significant at (*p* ≤ 0.01) and (*p* ≤ 0.05), respectively. Each value is a mean (± SE) of three replicates.

**Fig. 5:**
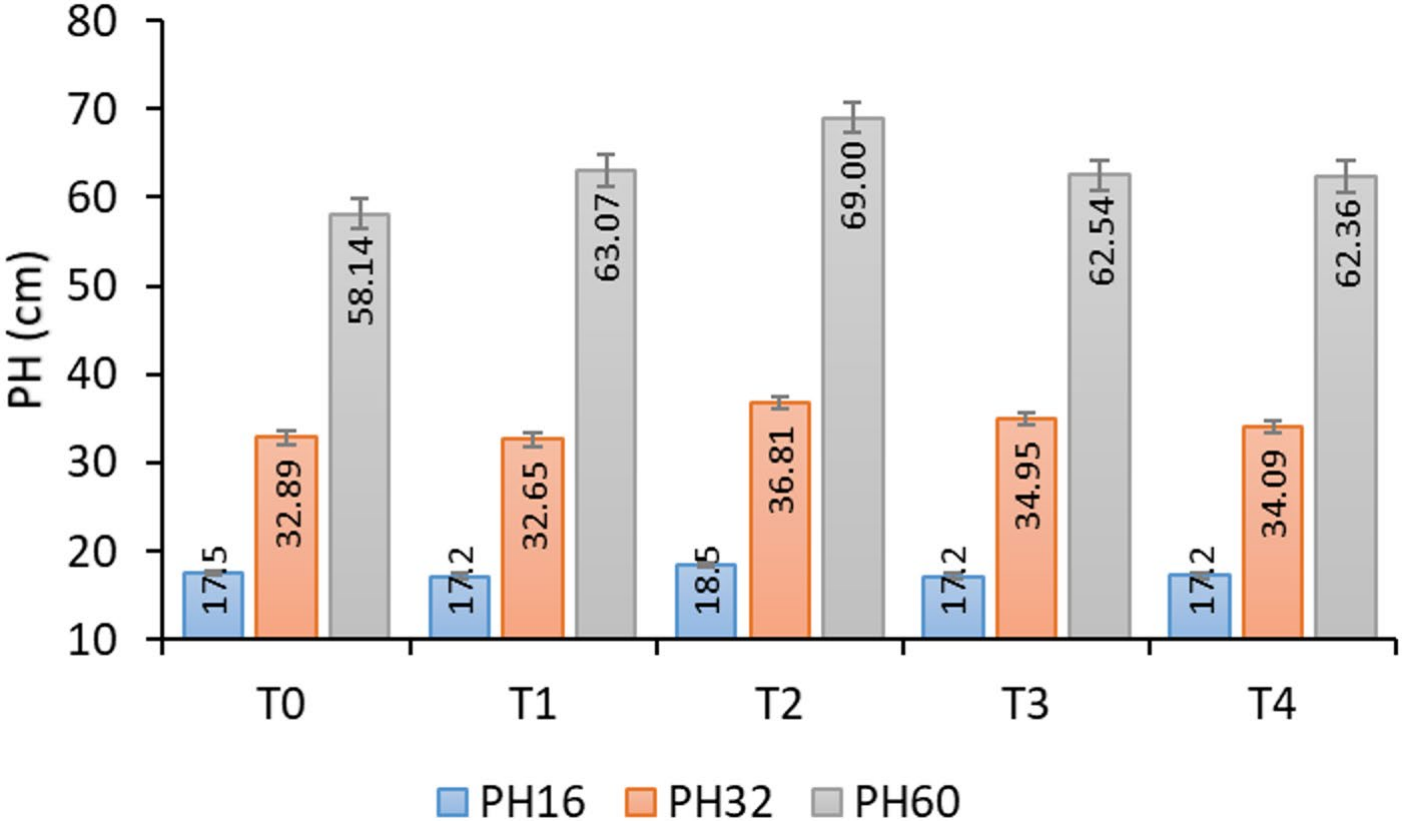
Effect of integrated organic-mineral fertilization treatments (T0–T4) on plant height (PH) at 16, 32 and 60 ADP of eggplant (*Solanum melongena* L.) grown in sandy soil. T0 represents the control group; T1–T4 represent increasing levels of organic-mineral integration. Error bars indicate the standard error (*n* = 3).

Data over two seasons presented in Fig. 5 shows that plant height increased progressively from PH16 to PH60 across all treatments. The lowest values were recorded under PH16, moderate values under PH32, and the highest under PH60. Treatment T2 consistently produced the greatest plant height, reaching the maximum value at PH60, while T0 showed the lowest overall performance. Treatments T3 and T4 exhibited similar intermediate responses.

#### Effects of SOFA individual or combined with mineral fertilizers of *S. melongena* on vegetative growth

Number of branches per plant^−1^, number of leaves per plant^−1^, fresh and dry weights and relative chlorophyll index are the most parameters that influence *S*. *melongena* growth at vegetative stage. Soil amendments significantly affected number of branches per plant^−1^, number of leaves per plant^−1^, fresh and dry weights and relative chlorophyll index in both growing seasons Table 4. The highest number of branches (4.43 and 4.57) produced from the T2 treatment in the first and second growing seasons, respectively Table 4. The application of SOFA individual or combined with mineral fertilizer, significantly increased number of branches in *S*. *melongena* plants in both growing seasons. The lowest value of number of branches was recoded with the application of control treatment and T4 in both growing seasons. As shown in Table 2. The average number of leaves per plant of treated plants with T2 was (47.42 and 47.66) in both growing seasons, respectively, which was significantly higher than that of the other fertilizer treatments (P < 0.05). However, the proportion of number of leaves per plant of T0 treatment was lower than that of T1 treatment (100% SOFA).

In the present study, at vegetative growth the minimum plant fresh weight (230.00 and 230.70 g) and plant dry weight (44.40 and 46.13 g) of *S*. *melongena* were observed with control treatment T0, while the moderate plant fresh weight (263.40 and 270.00 g) and plant dry weight (53.81 and 54.00 g) of eggplant were recoded with T3 in both growing seasons, respectively. In the current study, the maximum plant fresh weight (283.10 and 280.00 g) and plant dry weight (53.81 and 56.00 g) of eggplant were noted with 75% RD + 25% SOFA in both growing seasons, respectively Table 4. Consequently, the application of T1 significantly enhanced the relative chlorophyll index (SPAD). The highest SPAD reading values (63.47 and 63.00) in the first and second seasons, respectively, were obtained from the application of 100% SOFA Table 4.

**Table 4:** Effects of fertilization treatments on number of branches/plant (NB), number of leaves/ plant (NL), leaves fresh weight g (LFW), leaves dry weight g (LDW) and relative chlorophyll index (SPAD) in the cultivation of *Solanum melongena* in both seasons.

Treatments	NB	NL	LFW	LDW	SPAD
2023					
T0	3.57 ± 0.09	40.67 ± 0.44	230.00 ± 5.00	44.40 ± 0.28	62.20 ± 0.39
T1	4.10 ± 0.06	43.83 ± 0.67	267.90 ± 3.36	51.27 ± 0.41	63.47 ± 1.42
T2	4.43 ± 0.23	47.42 ± 0.46	283.10 ± 0.06	53.81 ± 0.39	62.95 ± 0.58
T3	4.34 ± 0.09	43.17 ± 0.17	263.40 ± 5.63	50.75 ± 0.41	57.84 ± 0.61
T4	3.95 ± 0.05	42.00 ± 0.00	263.40 ± 2.90	49.74 ± 0.67	60.08 ± 0.82
Significant	**	**	**	**	**
LSD_**0.05**_	0.39	1.32	12.65	1.51	2.66
2024					
T0	3.51 ± 0.00	41.03 ± 0.44	230.70 ± 0.67	46.13 ± 0.13	61.00 ± 0.00
T1	4.13 ± 0.01	44.68 ± 0.67	267.00 ± 0.53	53.40 ± 0.31	63.00 ± 0.00
T2	4.57 ± 0.03	47.68 ± 0.05	280.00 ± 0.00	56.00 ± 0.00	61.24 ± 0.33
T3	4.28 ± 0.01	43.29 ± 0.17	270.00 ± 0.00	54.00 ± 0.00	59.00 ± 0.00
T4	4.04 ± 0.03	42.14 ± 0.05	265.00 ± 0.00	53.00 ± 0.00	60.67 ± 0.33
Significant	**	**	**	**	**
LSD_**0.05**_	0.06	1.33	2.35	0.46	0.47

T0: Control; T1: 100% SOFA; T2: 75% RD + 25% SOFA; T3: 50% RD + 50% SOFA; T4: 25% RD + 75% SOFA. * and **, Significant at (*p* ≤ 0.01) and (*p* ≤ 0.05), respectively. Each value is a mean (± SE) of three replicates.

**Fig. 6:**
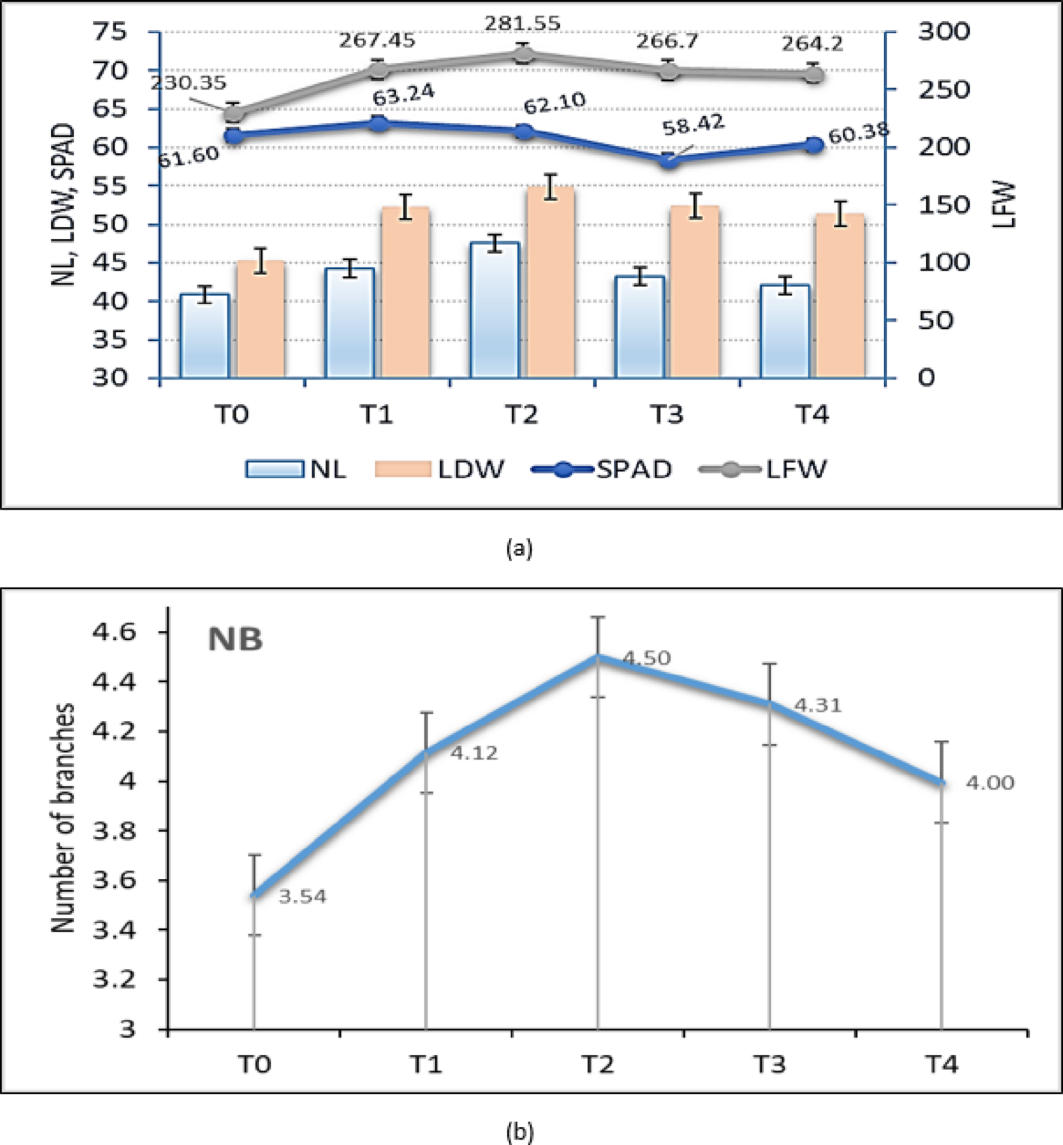
(**a**) Effects of fertilization treatments on number of leaves/plant (NL), leaves fresh weight g (LFW), leaves dry weight g (LDW) and relative chlorophyll index (SPAD) in the cultivation of Solanum melongena on average of both seasons. T0 represents the control group; T1–T4 represent increasing levels of organic-mineral integration. Error bars indicate the standard error (*n* = 3). (**b**) Effect of integrated organic-mineral fertilization treatments (T0–T4) on number of branches per plant (NB) of eggplant (*Solanum melongena* L.) grown in sandy soil. T0 represents the control group; T1–T4 represent increasing levels of organic-mineral integration. Error bars indicate the standard error (*n* = 3).

The Fig. 6a,b shows the effects of fertilization treatments in the combined seasons on NL, LDW, SPAD, LFW, and NB. Overall, T2 recorded the highest values for most parameters, including NL, LDW, and LFW, indicating superior vegetative performance. SPAD values also peaked at T1 and T2, with T2 showing strong chlorophyll content compared to other treatments. In contrast, T0 exhibited the lowest values across most traits. LFW followed a similar trend, increasing from T0 to T2, then slightly declining at T3 and T4. In summary, T2 was the most effective treatment in enhancing leaf growth, biomass accumulation, and chlorophyll content.

#### Effect of SOFA individual or combined with mineral fertilizer on fruit properties and yield of *S. melongena*

**Fig. 7:**
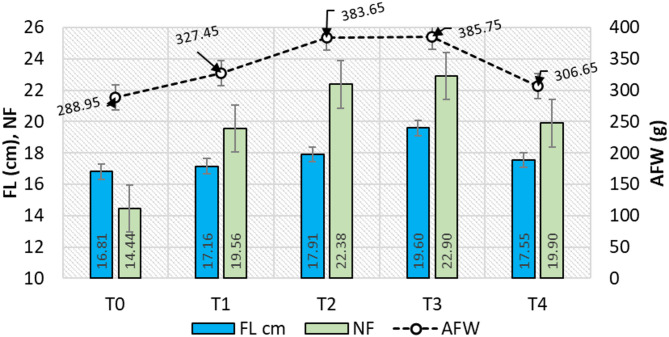
Effect of integrated organic-mineral fertilization treatments (T0–T4) on fruit length cm (FL), number of fruits per plant (NF), and average fruit weight g (AFW)) of eggplant (*Solanum melongena* L.) grown in sandy soil. T0 represents the control group; T1–T4 represent increasing levels of organic-mineral integration. Error bars indicate the standard error (*n* = 3).

As shown in Table 5. There was a significant difference in fruit diameter cm, and fruit length cm, and there was a significant difference in number of fruits per plant, average fruit weight g and total fruit yield per plant under different fertilization treatments. The average fruit diameter cm of T3 treatment (50% RD + 50% SOFA) was the widest, which reached (5.87 and 6.04 cm) in both growing seasons, respectively, whereas the average fruit diameter cm of T0 (recommended dose of N) treatment was the narrowest, which recoded (4.4.49 and 4.63 cm) in the first and second seasons, respectively. In addition, the maximum (19.19 and 20.00 cm) fruit length cm of *S. melongena* was in T3 which had a significant difference with respect to T0, while average fruit length of T0 treatment was the shortest, only (16.62 and 17.00 cm) in both growing seasons, respectively Table 5. Furthermore, treated plants with T3 increased the average number of fruits per plant by (65.36 and 60.87%) compared to T0. application of SOFA individual or combined with N had a positive effect on the number of fruits per plant in both growing seasons. In addition, the total fruit yield per plant in thrice harvests was found to be affected significantly with application of SOFA individual or combined with N fertilizer, the maximum fruit yield per plant (8830.00 g) in both growing seasons of eggplant crop were obtained with T3 treatments in the first and second seasons, respectively. The least total fruit yield per plant (4.330 and 4.002 kg plant^−1^) of *S*. *melongena* was produced from the T0 treatment. Whereas the T3 (8.360 kg plant^−1^) had a moderate total fruit yield per plant in the second season. As shown in Fig. 7 for combined two seasons, the integration of organic and mineral amendments significantly enhanced eggplant productivity in sandy soils, with the T3 treatment achieving a peak Total Fruit Yield (TFY) of 8.8 kg plant^−1^, a 111% increase over the control (T0: 4.18). This quantitative gain was mirrored by qualitative improvements in fruit diameter (FD), which reached a maximum of 5.96 cm in T3 compared to 4.56 cm in control. However, a performance decline in T4 (TFY: 6.12; FD: 5.29) indicates a specific optimal threshold for organic-mineral synergy, beyond which further integration becomes counterproductive. Ultimately, these results provide empirical evidence that balanced integrated fertilization effectively mitigates the fertility limitations of sandy substrates, outperforming both baseline and over-saturated nutrient regimes.

**Table 5:** Effects of fertilization treatments on fruit diameter cm, (FD), fruit length cm (FL), number of fruits per plant (NF), average fruit weight g (AFW), total fruit yield per plant kg (TFY), in the cultivation of *Solanum melongena* in both seasons.

Treatments	FD (cm)	FL (cm)	NF	AFW (g)	TFY (Kg)
2023					
T0	4.49 ± 0.01	16.62 ± 0.06	14.87 ± 0.90	290.90 ± 4.66	4.33 ± 325.46
T1	4.72 ± 0.01	16.99 ± 0.50	19.12 ± 0.58	325.20 ± 2.82	6.21 ± 147.33
T2	5.78 ± 0.04	17.82 ± 0.34	22.75 ± 0.76	387.30 ± 4.67	8.83 ± 437.67
T3	5.87 ± 0.02	19.19 ± 0.10	22.80 ± 0.13	387.50 ± 6.23	8.85 ± 153.67
T4	5.28 ± 0.03	17.73 ± 0.08	19.12 ± 0.73	293.30 ± 11.58	5.62 ± 432.47
Significant	**	**	**	**	**
LSD_**0.05**_	0.083	0.88	2.13	21.10	1025.80
2024					
T0	4.63 ± 0.01	17.00 ± 0.00	14.00 ± 0.00	287.00 ± 1.15	4.02 ± 16.17
T1	4.85 ± 0.01	17.33 ± 0.33	20.00 ± 0.00	329.70 ± 3.71	6.59 ± 74.24
T2	5.83 ± 0.03	18.00 ± 0.00	22.00 ± 0.00	380.00 ± 2.89	8.36 ± 63.51
T3	6.04 ± 0.04	20.00 ± 0.07	23.00 ± 0.33	384.00 ± 2.89	8.83 ± 122.69
T4	5.30 ± 0.03	17.37 ± 0.07	20.67 ± 0.33	320.0 ± 2.89	6.62 ± 122.69
Significant	**	**	**	**	**
LSD_**0.05**_	0.08	0.48	0.47	8.47	323.20

T0: Control; T1: 100% SOFA; T2: 75% RD + 25% SOFA; T3: 50% RD + 50% SOFA; T4: 25% RD + 75% SOFA. * and **, Significant at (*p* ≤ 0.01) and (*p* ≤ 0.05), respectively. Each value is a mean (± SE) of three replicates.

#### Effect of SOFA individual or combined with mineral fertilizers on the fruit quality of *S. melongena*

Data in Table 6. shows that dry matter %, total soluble solids TSS% and anthocyanin content greatly increased with application of SOFA individual or combined with mineral fertilizer. The highest significant values (11.21 and 11.90%) in the first and second growing seasons, respectively, for dry matter% were recorded in *S. melongena* treated with T2, followed by those treated with T3 (10.20 and 10.95%) in both growing seasons respectively. On the other hand, plants treated with T0 showed the lowest values of dry matter % (8.45 and 9.00%) in both growing seasons respectively, with significant differences among all treatments. In addition, the highest significant values of TSS% (7.83 and 7.92%) in both growing seasons respectively, were noted when plants treated with T3. While the lowest significant values (5.57 and 5.33%) of TSS% were showed in eggplant plants treated with T0. Table 6. Regarding anthocyanin content, data showed that the highest significant values (1.40 and 1.38 mg) in both growing seasons respectively, were obtained by using T2, followed by T3 (1.34 mg) in the first and second seasons respectively. While the lowest value (0.94 and 1.00 mg) in both growing seasons, was produced when plants treated with T0 Table 6.

**Table 6:** Effects of fertilization treatments on fruit dry matter (DM%) total soluble solids (TSS%) and anthocyanin content, in the cultivation of *Solanum melongena* in both seasons.

Treatments	2023			2024		
	DM%	TSS%	Anthocyanin	DM%	TSS%	Anthocyanin
T0	8.45 ± 0.21	5.57 ± 0.65	0.94 ± 0.00	9.00 ± 0.21	5.33 ± 0.09	1.00 ± 0.00
T1	9.30 ± 0.13	7.13 ± 0.19	1.07 ± 0.01	9.98 ± 0.06	7.20 ± 0.20	1.10 ± 0.00
T2	11.21 ± 0.11	7.62 ± 0.14	1.40 ± 0.03	11.90 ± 0.04	7.39 ± 0.04	1.38 ± 0.01
T3	10.20 ± 0.29	7.83 ± 0.04	1.34 ± 0.02	10.95 ± 0.10	7.92 ± 0.04	1.34 ± 0.02
T4	9.21 ± 030	7.35 ± 0.06	1.16 ± 0.00	9.49 ± 0.04	7.48 ± 0.04	1.24 ± 0.00
Significant	**	**	**	**	**	**
LSD_**0.05**_	0.70	0.98	0.05	0.35	0.34	0.03

T0: Control; T1: 100% SOFA; T2: 75% RD + 25% SOFA; T3: 50% RD + 50% SOFA; T4: 25% RD + 75% SOFA. * and **, Significant at (*p* ≤ 0.01) and (*p* ≤ 0.05), respectively. Each value is a mean (± SE) of three replicates.

**Fig. 8:**
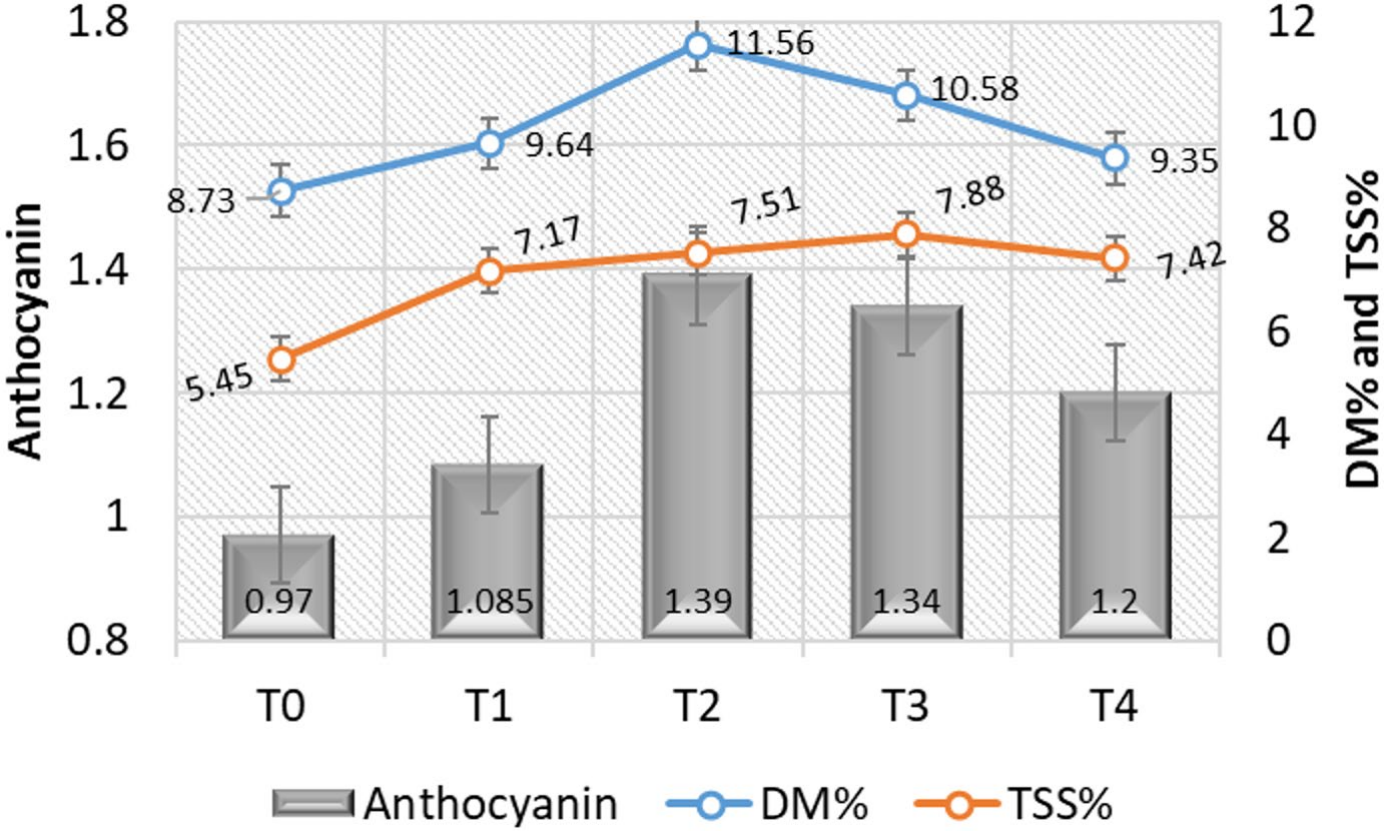
Effects of fertilization treatments on fruit dry matter (DM%) total soluble solids (TSS%) and anthocyanin content, in the cultivation of *Solanum melongena* on average of both seasons. T0: Control; T1: 100% SOFA; T2: 75% RD + 25% SOFA; T3: 50% RD + 50% SOFA; T4: 25% RD + 75% SOFA.

The results in Fig. 8 for the both seasons provide strong empirical evidence that while any level of organic-mineral integration outperforms the control (T0), the T2 and T3 treatments represent the optimal range for eggplant cultivation in sandy soils. Specifically, T2 optimizes physiological traits (Anthocyanin and DM%), while T3 maximizes marketable yield and sugar content (TSS%). The consistent drop-off across all metrics at T4 reinforces the need for a balanced, rather than a “more is better,” approach to amendment application.

#### Effect of SOFA individual or combined with mineral fertilizers on the nutrient’s uptake of *S. melongena*

During the current investigation, at harvest of eggplant crop, T3 and T4 treatments showed the most increased in the fruit content of N% (2.56%), P% (0.35%), and K% (2.00%), in the first growing season. While the lowest significant values were produced from plants treated with T0 for N% (2.10%) and K% (184%), in addition T3 (0.19%) for K% percentage in plant tissues. In the second growing season, the highest values for NPK % in eggplant fruit were observed when planted treated with T1 (2.72%), T4(0.40%) and T2(2.13%), respectively. T0 and T3 treatments were noted that they had a most significant increase (3.46%) of NPk % leaves contents Tables 7 and 8. On the other hand, the NPK% contents in eggplant leaves, the values (3.46 and 3.66%) of N% were increased significantly in both growing seasons respectively, in plants treated with 100% SOFA T0 Table 6. In addition, the lowest value (2.41 and 3.46) was produced from T3 and T4 treatments in the first and second seasons respectively. While T3 was found to be superior for P% in plant leaves (0.31 and 0.39%) in both growing seasons. In addition, the lowest values of that trait were produced from plants treated with T1 and T4 (0.23 and 0.34%) in the first and second seasons respectively. The content of K% in the plant tissues treated with T3 (2.17 and 2.28%) were recoded greater than the T0 (1.93 and 2.09%) in both growing seasons. Tables 7, 8.

**Table 7:** Effects of fertilization treatments on N%, P%, and K% in fruit (F), in the cultivation of *Solanum melongena* in both seasons.

Treatments	2022/2023			2023/2024		
	*N*% F	*P*% F	K% F	*N*% F	*P*% F	K% F
T0	2.10 ± 0.05	0.24 ± 0.02	1.84 ± 0.02	2.29 ± 0.03	0.39 ± 0.01	1.98 ± 0.04
T1	2.32 ± 0.04	0.24 ± 0.02	1.91 ± 0.02	2.72 ± 0.02	0.36 ± 0.01	2.02 ± 0.02
T2	2.11 ± 0.05	0.19 ± 0.01	1.92 ± 0.04	2.35 ± 0.01	0.29 ± 0.02	2.13 ± 0.01
T3	2.56 ± 0.10	0.30 ± 0.00	1.98 ± 0.03	2.52 ± 0.04	0.34 ± 0.01	2.12 ± 0.02
T4	2.45 ± 0.02	0.35 ± 0.02	2.00 ± 0.04	2.63 ± 0.01	0.40 ± 0.02	2.08 ± 0.01
Significant	**	**	**	**	**	**
LSD_**0.05**_	0.18	0.05	0.10	0.08	0.04	0.08

T0: Control; T1: 100% SOFA; T2: 75% RD + 25% SOFA; T3: 50% RD + 50% SOFA; T4: 25% RD + 75% SOFA. * and **, Significant at (*p* ≤ 0.01) and (*p* ≤ 0.05), respectively. Each value is a mean (± SE) of three replicates.

**Table 8:** Effects of fertilization treatments on N%, P%, and K% in leaves (L), in thecultivation of Solanum melongena in both seasons.

Treatments	2022/2023			2023/2024		
	*N*% L	*P*% L	K% L	*N*% L	*P*% L	K% L
T0	2.88 ± 0.16	0.29 ± 0.02	1.93 ± 0.03	3.64 ± 0.05	0.35 ± 0.02	2.09 ± 0.07
T1	3.46 ± 0.02	0.23 ± 0.05	2.01 ± 0.07	3.66 ± 0.05	0.37 ± 0.01	2.11 ± 0.00
T2	3.30 ± 0.28	0.28 ± 0.02	2.09 ± 0.01	3.63 ± 0.04	0.36 ± 0.01	2.20 ± 0.04
T3	2.41 ± 0.54	0.31 ± 0.02	2.17 ± 0.04	3.60 ± 0.05	0.39 ± 0.01	2.28 ± 0.04
T4	3.14 ± 0.09	0.27 ± 0.01	2.09 ± 0.04	3.46 ± 0.04	0.34 ± 0.01	2.19 ± 0.04
Significant	**	**	**	**	**	**
LSD0.05	0.96	0.08	0.12	0.17	0.04	0.17

T0: Control; T1: 100% SOFA; T2: 75% RD+25% SOFA; T3: 50% RD+50% SOFA; T4: 25% RD+75% SOFA. * and **,Significant at (p ≤ 0.01) and (p ≤ 0.05), respectively. Each value is a mean (±SE) of three replicate.

**Fig. 9:**
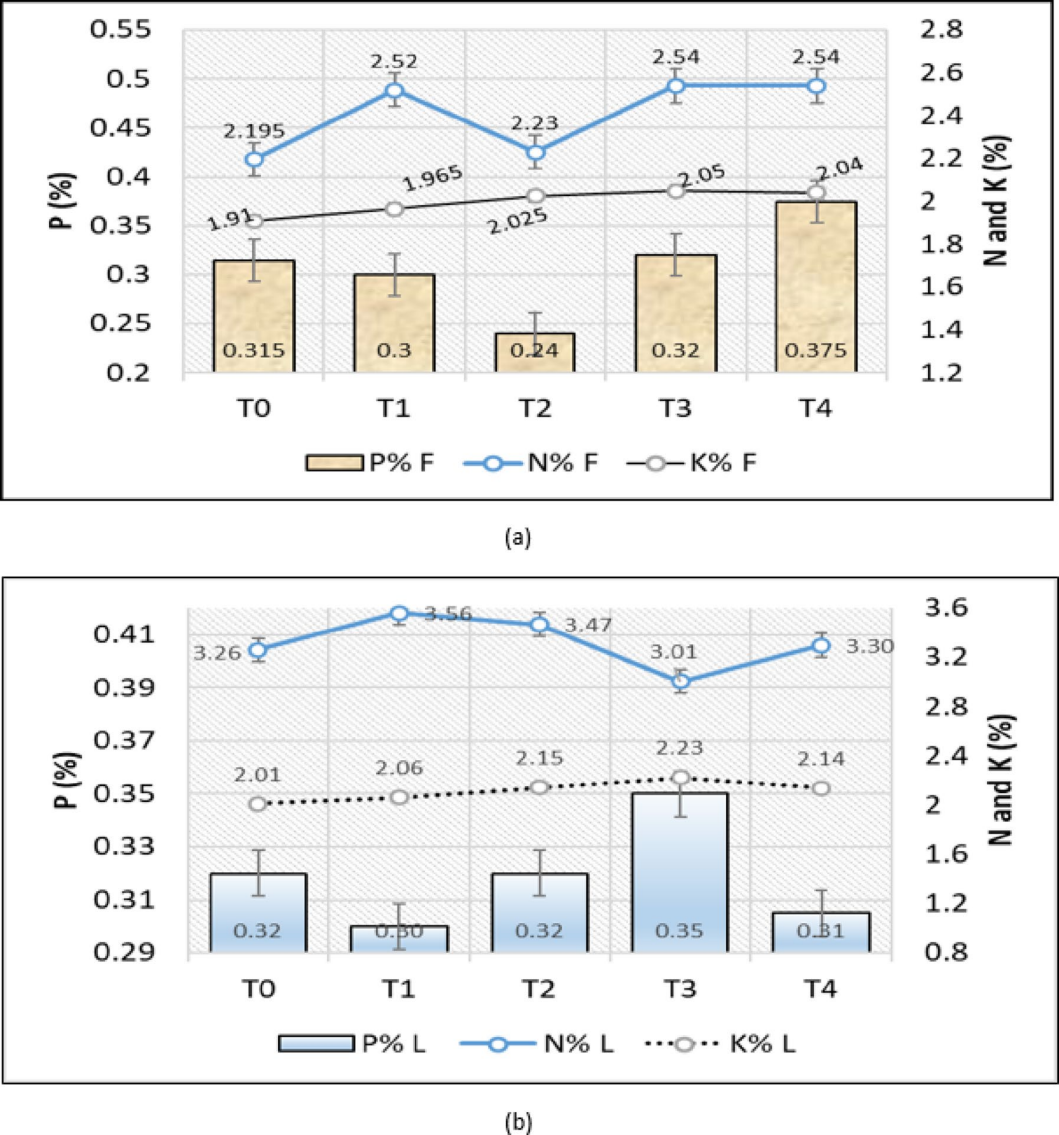
(**a**) Effects of fertilization treatments on N%, P%, and K% in fruit (F), in the cultivation of *Solanum melongena* on average of both seasons. (**b**) Effects of fertilization treatments on N%, P%, and K% in leaves (L), in the cultivation of Solanum melongena on average of both seasons.

The average results across both cultivation seasons in Fig. 9a,b demonstrate that integrated organic-mineral fertilization significantly modulates the nutrient partition and accumulation within the fruit and leaf tissues of *Solanum melongena L*. In the fruit, nitrogen (N) concentration peaked at 2.54% under both the T3 and T4 regimes, while phosphorus (P) attained its maximum value of 0.375% at the highest integration level (T4). Potassium (K) in the fruit exhibited a consistent upward trend, reaching an optimal concentration of 2.05% in the T3 treatment. Conversely, leaf nutrient dynamics revealed a distinct allocation pattern, with leaf nitrogen reaching its zenith at T1 (3.56%), whereas leaf phosphorus and potassium reached their highest recorded values of 0.35% and 2.23%, respectively, in the T3 treatment. These divergent accumulation patterns suggest that while early-stage integration favors vegetative nitrogen storage, the T3 threshold represents the most effective balance for maximizing the translocation of essential macronutrients to both the photosynthetic apparatus and reproductive sinks in sandy soil environments.

#### Effect of SOFA individual or combined with mineral fertilizers on soil chemical properties

At harvesting of *S. melongena*, T4 (25% RD + 75% SOFA), and T3 (50% RD + 50% SOFA) showed the most significantly increased in EC in both growing seasons respectively. The value of the soil EC treated with 100% SOFA were reduced EC by (21.00, 64.00 and 72.00%), and (70.00, 94.00, and 72.00%), compared to T2, T3 and T4. While the values were greater than T0. in the first season and second season respectively Table 9.

The results showed that application of SOFA individual or combined with different mineral fertilizer significantly changed pH, CaCo_3_ and organic matter (OM), of the soil used for growing eggplant in both growing seasons. The pH (8.41, 8.08, 8.09, and 8.24) was more alkaline for T1, T2, T3, and T4 respectively, in the first season as compared with T0 (7.53). While in the second season, the pH (8.31, 8.23, 8.09, and 8.23) for T1, T2, T3, and T4 respectively, was less alkaline compared to T0 (8.58) Table 6. In addition, soil CaCo_3_ content was decreased gradually, the most decreased values (6.51 and 6.42) were observed in plants treated with T3 in both growing seasons respectively. The soil organic matter was greater than T0 (0.53 and 0.58), in both seasons respectively, the contents of OM in the soil treated T2 (0.83) in the first season, and T3 (0.80) in the second season Table 9. On the other hand, the contents of available N, P and K in the soil treated with SOFA individual or combined with different ratios of mineral fertilizers were produced greater than the control soil. The higher values of available N (175.70 and 225.00 mg/kg) P (45.45 and 87.48),with and K (285.70 and 303.80 mg/kg) with T2 was obtained with T2 and T3, T2 and T1 and T3, T2 in the first and second season respectively. While the lowest values for available N (57.74 and 111.80 mg/kg), P (26.82 and 28.17 mg/kg), and K(182.00 and 208.00 mg/kg) were produced from plants treated with T0. Table 10.

**Table 9:** Effect of fertilization treatments on soil electric conductivity (EC), hydrogen (pH), calcium carbonate (CaCo_3_), soil organic matter (OM%), in the cultivation of Solanum melongena in both seasons.

Treatments	EC	pH	CaCo_3_		OM
2023					
T0	0.51 ± 0.02	8.53 ± 0.98	7.58 ± 0.08		0.53 ± 0.06
T1	0.76 ± 0.13	8.41 ± 0.05	7.35 ± 0.33		0.75 ± 0.10
T2	0.92 ± 0.05	8.08 ± 0.04	6.69 ± 0.42		0.83 ± 0.06
T3	1.25 ± 0.19	8.12 ± 0.12	6.51 ± 0.13		0.66 ± 0.10
T4	1.31 ± 0.17	8.24 ± 0.06	8.07 ± 0.14		0.52 ± 0.04
Significant	**	**	**		**
LSD_0.05_	0.41	1.40	0.81		0.24
2024					
T0	0.54 ± 0.01	8.58 ± 0.04	6.96 ± 0.03		0.58 ± 0.01
T1	0.64 ± 0.02	8.31 ± 0.01	6.92 ± 0.08		0.69 ± 0.02
T2	1.09 ± 0.01	8.23 ± 0.01	6.86 ± 0.02		0.73 ± 0.02
T3	1.24 ± 0.02	8.09 ± 0.01	6.42 ± 0.04		0.80 ± 0.01
T4	1.10 ± 0.01	8.23 ± 0.02	7.73 ± 0.06		0.60 ± 0.00
Significant	**	**	**		**
LSD_0.05_	0.05	0.09	0.15		0.04

T0: Control; T1: 100% SOFA; T2: 75% RD + 25% SOFA; T3: 50% RD + 50% SOFA; T4: 25% RD + 75% SOFA. * and **, Significant at (*p* ≤ 0.01) and (*p* ≤ 0.05), respectively. Each value is a mean (± SE) of three replicates.

**Table 10:** Effect of fertilization treatments on soil available N mg/kg, P mg/kg, and K mg/kg, in the cultivation of *Solanum melongena* in both seasons.

Treatments	2023			2024		
	*N*	*P*	K	*N*	*P*	K
T0	57.74 ± 1.60	26.82 ± 1.71	182.20 ± 3.65	111.80 ± 0.74	28.17 ± 1.04	208.60 ± 4.14
T1	94.73 ± 2.88	45.45 ± 2.07	219.60 ± 12.70	135.80 ± 2.29	68.17 ± 2.64	234.40 ± 2.71
T2	175.70 ± 4.17	43.67 ± 1.27	285.70 ± 10.26	196.50 ± 5.74	61.97 ± 1.02	303.80 ± 6.77
T3	112.70 ± 1.55	42.34 ± 2.23	240.90 ± 2.67	255.00 ± 13.34	87.48 ± 2.57	250.70 ± 5.38
T4	113.10 ± 2.43	34.8 ± 0.30	201.80 ± 6.37	166.70 ± 6.79	66.62 ± 1.08	229.60 ± 2.67
Significant	**	**	**	**	**	**
LSD_**0.05**_	8.53	5.25	25.51	22.85	5.79	14.83

T0: Control; T1: 100% SOFA; T2: 75% RD + 25% SOFA; T3: 50% RD + 50% SOFA; T4: 25% RD + 75% SOFA. * and **, Significant at (*p* ≤ 0.01) and (*p* ≤ 0.05), respectively. Each value is a mean (± SE) of three replicates.

**Fig. 10:**
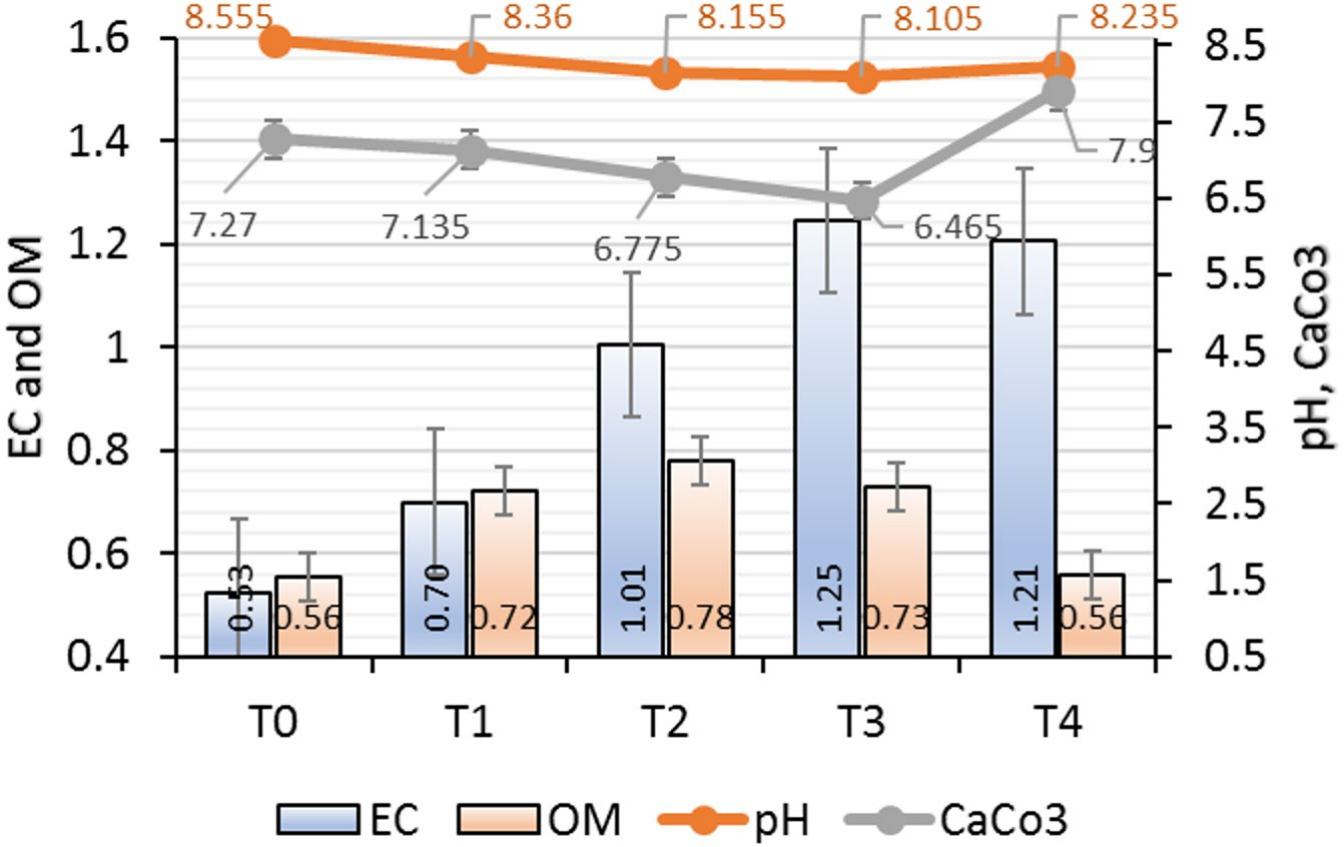
Effect of fertilization treatments on soil electric conductivity (EC), hydrogen (pH), calcium carbonate (CaCo3), soil organic matter (OM%), in the cultivation of Solanum melongena on average of both seasons.

**Fig. 11:**
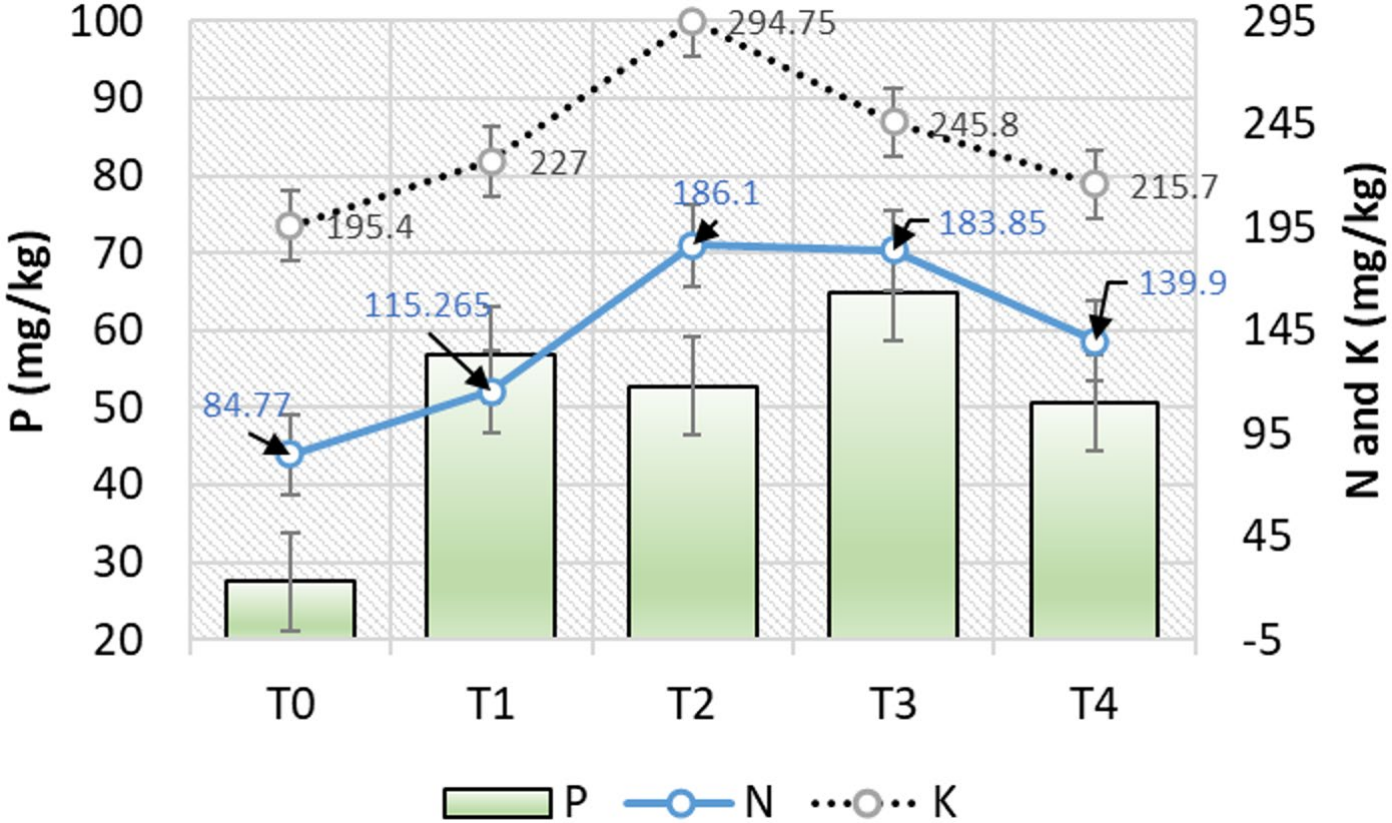
Effect of fertilization treatments on soil N mg/kg, P mg/kg, and K mg/kg, in the cultivation of Solanum melongena on average of both seasons.

On average across both cultivation seasons, as shown in Figs. 10 and 11 the integrated fertilization treatments significantly enhanced soil chemical properties and nutrient availability, which directly correlated with improved crop performance. Soil electrical conductivity (EC) and organic matter (OM%) reached their highest levels under the T3 treatment at 1.25 and 0.73%, respectively, whereas soil pH and CaCO_3_ exhibited a general decline as integration levels increased, reaching minimums of 8.105 and 6.465 in T3. This shift in the soil environment facilitated a substantial increase in available macronutrients, with soil nitrogen (N) peaking at 186.1 mg/kg in T2 and soil phosphorus (P) reaching its maximum of 65 mg/kg in T3, while soil potassium (K) attained a zenith of 294.75 mg/kg in T2. These soil-level improvements supported the previously noted peaks in fruit yield and nutrient accumulation, confirming that the T3 threshold provides the most favorable physicochemical environment for maximizing nutrient availability and uptake in sandy substrates.

#### Economic evaluation

A comparison of fertilizer type treatments as an economic evaluation of eggplant output is presented in Table 11. Nonetheless, the results were gross return per hectare, which was determined by multiplying “yield × price of eggplant.” Gross return/ha − Total cost/ha” was used to compute the net return. “Gross return/Total cost” was used to compute the investment factor (IF). The T0 treatment (100% NH₄NO₃/ha) had the lowest cost (177423.87 L.E.), while the T1 treatment (100% SOFA/ha) had the highest cost (242098.5 L.E.). The average total cost of producing one feddan of eggplant in Egypt is estimated to be about 80850.36 L.E., i.e., 192423.87 L.E. for hectare.

T3 had the highest gross returns per hectare, followed in descending order by T2, T1, and T4. Additionally, the T0 treatment produced the lowest net return, while the T3 treatment produced the highest net return (827198.8134), followed by T2 with no significant variations between them. T2 and T3 treatments, on the other hand, had the largest investment factor (about 5).

**Table 11:** Economic analysis and benefit cost ratio of different fertilization treatments of the experiment.

Treatments	Total yield^1^	Gross return^2^	Total cost^3^	Net return^4^	IF^5^	Order
T0	99.365	496,825	192423.8731	304401.1269	2.58	5th
T1	152.32	761,600	257098.5	504501.5	2.96	4th
T2	204.561	1,022,805	208592.5299	814212.4701	4.90	1st
T3	210.392	1,051,960	224761.1866	827198.8134	4.68	2nd
T4	145.656	728,280	240079.8433	488200.1567	3.03	3rd

^1^Total yield (ton/ha) as average of two seasons.

^2^Gross return as total yield (ton/fed) × 5000 L.E /ton

^3^Total cost: 16925.37 L.E. of 1128.358 kg NH_4_NO_3_/ha (100%RD, 378 N, kg NH_4_NO_3_=15 L.E.); 81,600 L.E. of SOFA/ha (100%RD, 378 N, liter SOFA = 8.5 L.E.) and 175498.5 L.E. for Seeds/seedlings, P and k fertilizers, pesticides, labor, irrigation/ha

^4^Net return = Gross Return-Total cost

^5^IF: The investment factor (The benefit-cost ratio) was calculated by dividing the gross income by total cost

When compared to individual applications such as standard chemical forms or other fertilizer treatments, the prior results generally showed that fertilizing eggplant with T2 (25% SOFA) and T3 (50% SOFA) fertilizers was the best treatment for yield, quality, and nutrient uptake. Additionally, compared to both individual treatments (T0 and T1), fertilizer applications such as T2 and T3 maximize production profitability. Treatment T2 was shown to be the most profitable and economically efficient alternative for production based on net return, investment factor (IF), and overall ranking, followed by T3. Due to its lower manufacturing cost in relation to return, T2 demonstrated higher economic efficiency even if T3 had the largest net return. Additionally, using the treatments (T2 and T3) may lessen the load or demand on chemical fertilizers, improve the effectiveness of applied N-fertilizers, and ultimately lower the added rate of N. As a result, it is now crucial to apply both chemical and organic fertilizers as mixed fertilizers.

#### Pooled analysis

For most vegetative, physiological, and yield-related variables, the pooled analysis of the two experimental seasons showed significant differences between treatments (Supplementary S3, S4). When compared to the control, plant growth parameters significantly improved with the applied treatments. Over the course of the growth period, plant height climbed gradually. 60 days after transplanting, T2 produced the tallest plants (69.00 cm), followed by T1 and T3. The control treatment recorded the lowest results. Improved food availability and higher metabolic activity, which encourage cell division and elongation processes, may be responsible for the increased vegetative development under T2^42^.

Additionally, branching and leaf development were much improved. T2 had the greatest number of branches (4.50) and leaves (47.55), indicating enhanced canopy development and vegetative vigor. Increased leaf area can improve assimilation production and photosynthetic capability, which eventually supports increased biomass accumulation and yield formation. Similar trends were seen in leaf biomass, with T2 having the largest leaf dry weight (54.91 g) and fresh weight (281.55 g). Improved photosynthate uptake and partitioning within plant tissues is suggested by the increase in dry matter percentage under the same treatment. In vegetable crops, improved biomass accumulation under nutrient-enriched circumstances has been extensively documented and is linked to increased physiological activity.

Additionally, quality characteristics responded favorably to the treatments. The highest total soluble solids (7.88%) were produced by T3, suggesting better fruit storage of carbohydrates. Anthocyanin content, on the other hand, was highest under T2, indicating increased secondary metabolite synthesis. Fruit quality and antioxidant capability may be enhanced by anthocyanins, which are known to rise under better physiological conditions^43^.

## Discussion

The present study clearly demonstrates that integrating sugarcane-derived organic amendments (SOFA) with mineral nitrogen fertilizer significantly enhances the productivity, quality, and soil health of *Solanum melongena* cultivated in sandy soils. The superiority of integrated treatments (T2 and T3) over sole mineral or organic applications highlights the importance of synergistic nutrient management strategies. Reducing the application of 25% N and replacing it with SOFA will produce the highest plants compared to other treatments. Tables 3 and 4. Shows that the application of 75% N and 25% SOFA fertilizer will produce the tallest plants than the application other treatments at 14, 32 and 60 days after transplanting in both growing seasons.

This finding could be related to the critical role of nitrogen in plants, which can be found in nucleic acids, co-enzymes, and proteins. in addition, the observed increase in vegetative growth under T2 (75% *N* + 25% SOFA) can be attributed to improved nitrogen use efficiency and balanced nutrient availability. Nitrogen plays a fundamental role in chlorophyll synthesis, protein formation, and enzymatic activity, thereby enhancing photosynthetic capacity and biomass accumulation. The addition of SOFA likely improved microbial activity and soil structure, facilitating gradual nutrient release and reducing nutrient losses through leaching, which is particularly critical in sandy soils.

Vos et al.^44^, indicted that the high N content in plants, produced the maximal number of leaves, wider plant size and increased plant photosynthesis. The photosynthate produced can be used as a raw resource for further plant growth and development. According to Porra; Kumar et al.; Syarief^45–47^ it may be possibly due to the maximum uptake of N, P and K by eggplant plants. The increasement of crop growth could be attributed to the role of K in the nutrient and sugar translocation in the plants and turgor pressure in the plant cells. It is also involved in cell enlargement and in triggering young tissue or meristematic growth. According to Naik and Rao^48^, lower organic fertilizer treatments resulted in the greatest vegetative growth characteristics such as plant height, root length, dry weight, chlorophyll content, and leaf area index of sunflower (*Helianthus annuus* L.). The results showed that the vegetative growth of *S*. *melongena* decreased at higher treatments (100% SOFA, and 75% SOFA + 25% N). This result agrees with those of^44–48^. In addition, T2 was the superior treatment for number of branches, number of leaves, and fresh leaves and dry weights, while 100% SOFA was produced the highest values for relative chlorophyll content, in both growing seasons. Which may be due to the existence of additional contents of the nutrients, amino acids, cytokines, and trace elements in the higher SOFA application, the plant height, number of branches, number of leaves per plant, leaf fresh and dry weights and chlorophyll content were converted into net growth, which could more clearly reflect the effects of different fertilizer treatments on eggplant plant growth. Furthermore, it may be due to Fe, Mg and Mn contents in the sugarcane byproducts, which resulted in chlorophyll synthesis^44,48,49^. These findings are consistent with the study conducted by^47–50^. In the present study, quality parameters of *S. melongena* i.e. DM, TSS, and anthocyanin content were significantly increased in the fruit treated with T2 and T3, it may be due to application of SOFA increased the growth and yield of eggplants which reflected to increasing in dry matter, total soluble solids and anthocyanin content^51,52^.

Data presented in Table 5 clearly showed that all fruit properties and yield were significantly affected by fertilizer treatments as compared to the control treatment. It’s clear that parameters highest values were significant at Table 5; Fig. 7 which produced when plants treated with 50% *N* + 50% SOFA. This increased may be attributed to the SOFA role, which contains growth factors and a relatively larger proportion of cytokinins, free amino acids, several vitamins as well as elements (Na, Ca, Fe, Mg, K, P, S, Zn and Si), and organic compounds, which play an essential role and have a stimulatory influence on cell division and enlargement features. According to Kumar and Chopra^46^. The main factor affecting *S*. *melongena* yield was not the difference in individual fruit fresh weight of eggplants resulted from application of different fertilizer treatments, but the amount of harvested eggplant under different fertilizer treatments. The yield of T0 was only 4.330 and 4.020 Kg plant^−1^ in both growing seasons respectively, while the SOFA treatment recorded 6.210 and 6.590 kg plant^−1^ in both seasons respectively. In addition, the T3 treatment produced the highest fruit yield, which reached 8.830 kg plant^−1^ in the first and second seasons.

The function of K, Fe, Mg, and Mn during fruiting is critical and relates to the formation of chlorophyll, which increases crop yield^50,51^, also, improves the yield of eggplants as obtained by^54,55^.

This finding is consistent with the findings of^56^ on sugar beet, who found that molasses applications significantly boosted root yield and quality compared to the control, whereas soil applications were more effective than foliar applications for all parameters investigated. As a result, molasses can be utilized efficiently to boost sugar beet yield and quality.

The demand for NPK nutrients in *S*. *melongena* was studied at various stages of growth, and the dose of N had a greater influence on leaf area than the dose of K. Furthermore, the stem diameter, plant height, and yield were all significantly affected^55–57^.

According to Maghfoer et al.^58^, a treatment of 75% urea + 25% goat dung improved plant growth and yielded the maximum fruit yield (48.7 t ha) when compared to a mixture of other different fertilizer treatments and 100% urea. This is since the plant’s requirements for each nutrient vary depending on the availability of nutrients in the soil. In general, maximum productivity is related to all plant conditions as well as nutrient availability in the soil. It is said to be optimal if all the constituents are present in sufficient quantities. A nutrient deficiency or excess might affect the effectiveness of other nutrients^59,60^.

The application of treated molasses (SOFA) as soil amendments significantly affected the concentrations of the N, P, and K in the eggplants leaves and fruit Table 5. The highest values of the N, P, and K in the leaves of eggplant (2.56, 0.35, and 2.00%) resulted from the application of T3 and T4 (Table 7) in the first season. While, in the second season the concentrations of N, P and K in eggplants fruit were (2.72, 0.40 and 2.13%), when plants treated with T1, T4, and T2 respectively. Furthermore, the application of T1 and T3 treatments drastically increased the average content of N, P and K in the leaves of eggplants (3.46 and 3.66%), (0.31 and 0.39%) and (2.17 and 2.28%) in both seasons respectively Table 8. Related to this^56,57^, argued that increasing the supply of N can encourage the growth of both shoot and root systems within a specific range, but often promotes the growth of shoot more than the root system, resulting in a fall in root/shoot ratio with increased N application. Furthermore, the use of 30% N fertilizer in combination with organic source is an excellent nutritional management method for maintaining N absorption and crop productivity, reducing N loss, and increasing soil fertility^61,62^ Table 8 and Fig. 9a,b.

During the current study, the results showed that application of SOFA individual or combined with mineral fertilizer of N significantly changed EC, pH, CaCo_3_,N, P and K of the soil which was used for the growing of eggplants in both the first and second seasons. Table 9. The EC value was raised (0.76 and 0.64 ds/m) with application of 100% SOFA compared to (0.51 and 0.54 dS/m) 100% mineral N in both seasons respectively, but the increase in EC values is still the lowest in comparison with other fertilizers treatments. The accumulation of additional cations and anions in the soil suspension could explain these results^44^, The pH (8.41 and 8.31 in both seasons respectively) of the soil was found to be more alkaline with 100% SOFA treatment and it is likely due to the alkaline nature (pH 8.78) of SOFA, in the second season the pH of the soil was decreased gradually (8.31, 8.23, 8.09, and 8.23) with T1, T2, T3 and T4 respectively, in comparison with T0 (8.58). The pH of the soil is an important characteristic, according to several studies, because many nutrients are only available to plants within a specific pH range. A pH of 6.0–9.4 increases nutrient accessibility for plants, while a pH of less than 6.0 and greater than 8.8 restricts nutrient availability for plants^62,63^. During the current investigation, the pH of the soil ranged from 8.41 to 8.31 with 100% SOFA treatment, making the different soil nutrients available to the plants.

The data in Fig. 10 indicates that the contents of CaCO_3_ in the soil treated with 100% SOFA were produced (7.35 and 6.92%) less than 100% mineral N (7.58 and 6.96%) in both growing seasons of eggplant respectively. Such results may be due to increasing organic matter in the soil, which resulted in decreased in CaCO_3_ with application of organic fertilizers^64^.

The slightly higher soil electrical conductivity (EC) observed at higher SOFA application rates may be attributed to the release of soluble nutrients during the decomposition and mineralization of the organic constituents in the amendment. Similar increases in EC following the application of organic fertilizers or organic-based soil amendments have been reported in previous studies and are generally associated with improved nutrient availability rather than harmful salinity effects when EC values remain within the acceptable range for crop production^65^.

The significant increase in N, P and K the soil in response to the application of SOFA combined with (T2 and T3) in both seasons, in general, the increase in the total soil N content was associated with increasing organic fertilizer addition level Table 10. So, the maximum of total nitrogen (175.70 mg/kg) was produced with application of 25% SOFA + 75% mineral N and this due to, a major amount of the nitrogen (N) in SOFA is in organic form, which requires more time for decomposition to make the organic N molecules accessible and, subsequently, to release N from those molecules, reducing nitrogen loss by washing and uptake by plants^65,66^.In addition, in the both seasons, the maximum values of available soil P in most cases were found in the soil amended with 100% SOFA in the first season, 50% *N* + 50% SOFA in the second season. It might be due to, the amounts of P in SOFA fertilizer which helps to enhance the nutrient status including increase the availability of P by converting, also may be due to the cumulative effect of Inorganic and organic acids and CO_2_ produced, as well as the reduction in soil pH reduction, resulted in increasing the soil P availability for plants Fig. 11.

Generally, the available K of the soil increased with SOFA applicated alone or with mineral N could be due to the release of K during the mineralization of organic materials^44^. In addition, increasing available K in SOFA fertilizer which had many components such as mineral supplements, organic acids, active microorganisms help to enhance the release of K from soil minerals (mica and feldspar).

**Fig. 12:**
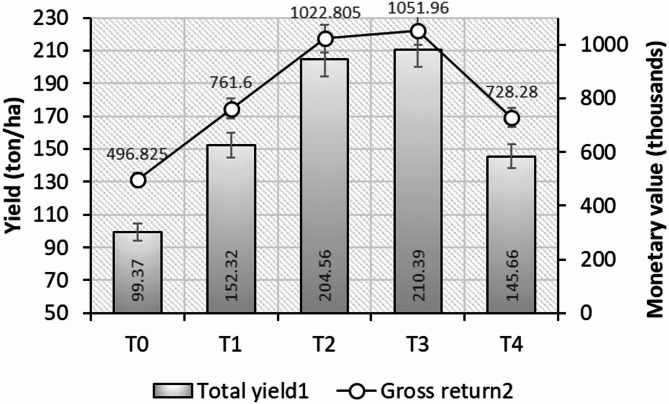
Monetary value advantage (thousand pounds L.E./ha) as affected by organic fertilizer treatments on average of two seasons.

The highest cash advantage (1051.96 thousand L.E.) was obtained from T3 (50% mineral *N* + 50% organic N), followed by T2 (1022.80 thousand L.E.), i.e., 75% mineral *N* + 25% organic N, which is greater than all the monetary values resulting from fruit yield, according to the obtained values shown in Table 11; Fig. 12. According to our findings, the organic fertilizer treatments may be crucial for conventional farming practices to achieve high yield and/or some of its key components. This confirms that reducing mineral fertilizer inputs while incorporating organic amendments is not only environmentally sustainable but also economically advantageous.

This study was conducted over a two season at one location and tested only one crop species and specific SOFA application rates. Long-term soil fertility, environmental effects and microbial analysis were not evaluated, which may limit the generalizability of the results and highlights the need for multi-location and multi-season validation.

## Conclusion

In conclusion, the present study provides the sugar cane processing byproduct molasses treated with a mixture of some elements in nanoparticles form as a powerful and cost-effective soil amendment with a great potential for sustainable production of crop plants in soils for conservation agriculture. Treated molasses is a promising organic soil amendment that could greatly improve the physical and chemical properties of the soil, organize nutrients and elements uptake, and ultimately impact the quality and biofortification of crop plants. also, this study concluded that SOFA boosted soil nutrients and influenced the agronomical characteristics eggplant in both seasons. The 75%*N* + 25%SOFA treatment resulted in the best agronomical performance and biochemical components such as dry matter, total soluble solids, and anthocyanin concentration of *S*. *melongena*. More research on the agronomic growth and changes in biochemical composition following SOFA treatments is needed under varied field conditions.

Also, this study concluded that SOFA boosted soil nutrients and influenced the agronomical characteristics of eggplant in both seasons. The 75%*N* + 25%SOFA treatment resulted in the best agronomical performance and biochemical components such as dry matter, total soluble solids, and anthocyanin concentration of S. melongena. More research on agronomic growth and changes in biochemical composition following SOFA treatments is needed under varied field conditions.

The integration of organic and mineral amendments significantly optimized the productivity and physiological quality of eggplant in sandy soil. The T3 treatment (75% Mineral + 25% Organic) emerged as the agronomic optimum, facilitating a 111% increase in Total Fruit Yield (8.84 t/ha) compared to the control, while maximizing sugar accumulation (7.88% TSS).

Physiological analysis revealed that moderate integration (T2) was particularly effective for enhancing Anthocyanin content (1.39 mg/100 g) and Dry Matter accumulation (11.56%), indicating a high degree of nutrient utilization efficiency. These improvements were directly supported by the transformation of the sandy substrate, specifically a 28% increase in Soil Organic Matter and a significant increase in available P and K.

Collectively, these findings demonstrate that integrated fertilization regimes effectively overcome the fertility constraints of sandy soils by enhancing both the physical yield and the chemical density of the fruit, without the need for excessive mineral supplementation.

## Supplementary Information

Below is the link to the electronic supplementary material.

Supplementary Material 1

## Data Availability

The datasets used and/or analyzed during the current study are available from the corresponding author on reasonable request.
