# Supplementary material for: Integrated application of sugarcane by-product-derived organic fertilizer (SOFA) and mineral nitrogen enhances yield, fruit quality, and soil properties of eggplant (Solanum melongena L.) in sandy soil conditions
Source: Sci Rep. 2026 Jul 7;16:20996. doi: 10.1038/s41598-026-60547-1 (PMC13341758; doi:10.1038/s41598-026-60547-1)
Supplement: Supplementary file 1 — Supplementary Material 1 [file 41598_2026_60547_MOESM1_ESM.docx]

**Table S1: The total compounds analysis in SOFA**

| RT | Compound Name | Area % | MF | Molecular Formula | Mol. Wt. | CAS # | Library |
| --- | --- | --- | --- | --- | --- | --- | --- |
| 4.04 | Cyclononanone, 2-methyl- | 1.09 | 635 | C10H15D3O | 157 | 32454-54-9 | WileyRegistry8e |
| 4.04 | 3-Azabutyl-1-ol, 4-cyclopropyl-3,3-dimethyl-, bromide | 1.09 | 660 | C8H18NO | 144 | NA | mainlib |
| 4.04 | Ethan imidothioic acid, 2-(dimethylamino)-[(methylamino)carbonyl]oxy-, methyl ester | 1.09 | 633 | C7H13N3O3S | 219 | 23135-20-0 | WileyRegistry8e |
| 4.04 | 2-(Methyl-D3)-cyclooctanone | 1.09 | 624 | C9H13D3O | 143 | 32454-51-6 | WileyRegistry8e |
| 4.04 | 4-Cyclopentene-1,3-diol-D2, trans- | 1.09 | 708 | C5H6D2O2 | 102 | 40524-90-1 | WileyRegistry8e |
| 4.24 | 2-Pentanone, 4-hydroxy-4-methyl- | 3.35 | 837 | C6H12O2 | 116 | 123-42-2 | WileyRegistry8e |
| 4.24 | 2-Pentanone, 4-hydroxy-4-methyl- | 3.35 | 844 | C6H12O2 | 116 | 123-42-2 | mainlib |
| 4.24 | 2-Pentanone, 4-hydroxy-4-methyl- | 3.35 | 816 | C6H12O2 | 116 | 123-42-2 | WileyRegistry8e |
| 4.24 | 2-Pentanone, 4-hydroxy-4-methyl- | 3.35 | 876 | C6H12O2 | 116 | 123-42-2 | replib |
| 4.34 | 2-Pentanone, 4-hydroxy-4-methyl- | 5.78 | 872 | C6H12O2 | 116 | 123-42-2 | mainlib |
| 4.34 | 2-Pentanone, 4-hydroxy-4-methyl- | 5.78 | 910 | C6H12O2 | 116 | 123-42-2 | replib |
| 4.34 | 2-Pentanone, 4-hydroxy-4-methyl- | 5.78 | 863 | C6H12O2 | 116 | 123-42-2 | WileyRegistry8e |
| 4.56 | 2-Nitrohept-2-en-1-ol | 4.89 | 652 | C7H13NO3 | 159 | 104313-51-1 | mainlib |
| 4.56 | 2-Nitro-2-hepten-1-ol | 4.89 | 652 | C7H13NO3 | 159 | NA | WileyRegistry8e |
| 4.56 | 1,3:2,5-Dimethylene-l-rhamnitol | 4.89 | 676 | C8H14O5 | 190 | NA | mainlib |
| 4.56 | Clasto-lactacystin δ-lactone | 4.89 | 667 | C10H15NO4 | 213 | 154226-60-5 | nist_msms |
| 4.56 | 3-Cyclopropyl-4-hydroxy-4,5,5-trimethyl-oxazolidin-2-one | 4.89 | 659 | C9H15NO3 | 185 | NA | mainlib |
| 4.94 | Benzene, 1-ethyl-3-methyl- | 2.44 | 879 | C9H12 | 120 | 620-14-4 | mainlib |
| 4.94 | Benzene, 1-ethyl-4-methyl- | 2.44 | 856 | C9H12 | 120 | 622-96-8 | WileyRegistry8e |
| 4.94 | Benzene, 1-ethyl-4-methyl- | 2.44 | 899 | C9H12 | 120 | 622-96-8 | WileyRegistry8e |
| 5.59 | 7,7-Dimethyl-tetracyclo[4.1.0.0(2,4).0(3,8)]heptane | 4.48 | 936 | C9H12 | 120 | 124345-62-6 | WileyRegistry8e |
| 5.59 | 7,7-Dimethylnorbornadiene | 4.48 | 915 | C9H12 | 120 | 68757-94-8 | WileyRegistry8e |
| 5.59 | Benzenepentanenitrile, α-methyl-, delta-oxo- | 4.48 | 934 | C12H13NO | 187 | 58422-86-9 | WileyRegistry8e |
| 5.59 | Benzenepentanenitrile, α-butyl-, delta-oxo- | 4.48 | 893 | C15H19NO | 229 | 58422-89-2 | WileyRegistry8e |
| 5.59 | Benzene, 1,3,5-trimethyl- | 4.48 | 769 | C9H12 | 120 | 108-67-8 | WileyRegistry8e |

***
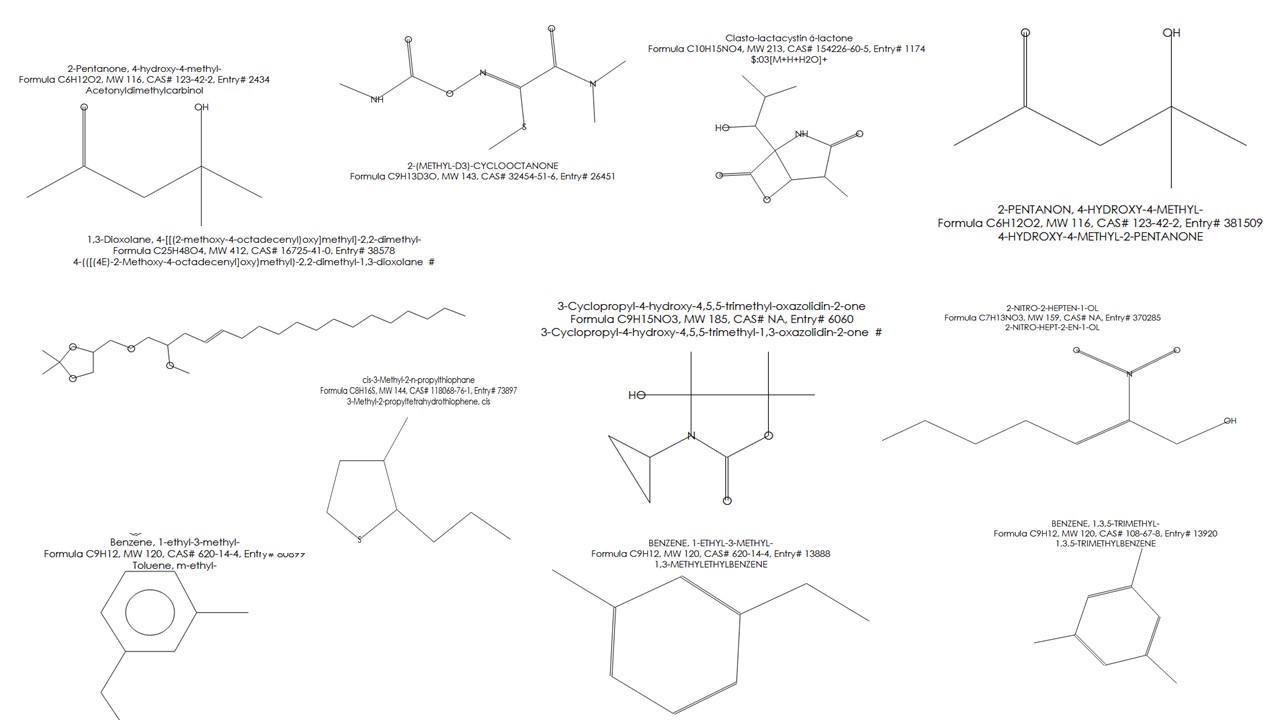
***

**Figure S1: Some compounds presented in SOFA**

**Table S2. Normality and homogeneity tests for pooled data across treatments**

| Trait | Shapiro–Wilk W | P-value | Levene’s F | P-value |
| --- | --- | --- | --- | --- |
| PH16 | 0.964 | 0.71 | 1.23 | 0.31 |
| PH32 | 0.951 | 0.56 | 1.08 | 0.37 |
| PH60 | 0.948 | 0.52 | 1.41 | 0.24 |
| DM% | 0.972 | 0.81 | 1.19 | 0.33 |
| TSS | 0.958 | 0.64 | 1.02 | 0.4 |
| Anthocyanin | 0.967 | 0.75 | 1.17 | 0.34 |
| NB | 0.953 | 0.59 | 1.21 | 0.32 |
| NL | 0.949 | 0.53 | 1.34 | 0.27 |
| LFW | 0.961 | 0.69 | 1.11 | 0.36 |
| LDW | 0.957 | 0.63 | 1.06 | 0.38 |
| SPAD | 0.968 | 0.76 | 1.14 | 0.35 |
| FD | 0.955 | 0.6 | 1.2 | 0.33 |
| FL | 0.952 | 0.57 | 1.25 | 0.30 |
| NF | 0.959 | 0.66 | 1.18 | 0.34 |
| AFW | 0.954 | 0.59 | 1.36 | 0.26 |
| TFY | 0.947 | 0.51 | 1.42 | 0.23 |

**Table S3** Effect of fertilization treatments on all studied traits on average of both seasons

| Parameters | T0 | T1 | T2 | T3 | T4 | LSD | F value | CV |
| --- | --- | --- | --- | --- | --- | --- | --- | --- |
| PH16 | 17.49 | 17.19 | 18.51 | 17.19 | 17.23 | 0.73 | 5.34 | 4.06 |
| PH32 | 32.89 | 32.65 | 36.81 | 34.95 | 34.09 | 0.95 | 42.61 | 2.78 |
| PH60 | 58.14 | 63.07 | 69.00 | 62.54 | 62.36 | 1.68 | 34.79 | 2.78 |
| DM% | 8.73 | 9.64 | 11.56 | 10.58 | 9.35 | 0.525 | 41.35 | 4.2 |
| TSS% | 5.45 | 7.17 | 7.51 | 7.88 | 7.42 | 0.66 | 14.41 | 5.88 |
| Anthocyanin | 0.97 | 1.09 | 1.39 | 1.34 | 1.20 | 0.04 | 92.63 | 1.58 |
| N% F | 2.20 | 2.52 | 2.23 | 2.54 | 2.54 | 0.13 | 6.18 | 4.8 |
| P% F | 0.32 | 0.30 | 0.24 | 0.32 | 0.38 | 0.045 | 5.01 | 7.07 |
| K% F | 1.91 | 1.97 | 2.03 | 2.05 | 2.04 | 0.09 | 4.53 | 3.36 |
| N% L | 3.26 | 3.56 | 3.47 | 3.01 | 3.30 | 0.565 | 3.01 | 10.66 |
| P% L | 0.32 | 0.30 | 0.32 | 0.35 | 0.31 | 0.06 | 1.94 | 9.25 |
| K% L | 2.01 | 2.06 | 2.15 | 2.23 | 2.14 | 0.145 | 3.62 | 5.56 |
| N | 84.77 | 115.27 | 186.10 | 183.85 | 139.90 | 15.69 | 32.8 | 7.96 |
| P | 27.50 | 56.81 | 52.82 | 64.91 | 50.71 | 5.52 | 23.74 | 7.66 |
| K | 195.40 | 227.00 | 294.75 | 245.80 | 215.70 | 20.17 | 21.63 | 6.55 |
| NB | 3.54 | 4.12 | 4.50 | 4.31 | 4.00 | 0.225 | 12.48 | 3.68 |
| NL | 40.85 | 44.26 | 47.55 | 43.23 | 42.07 | 1.325 | 18.67 | 2.15 |
| LFW | 230.35 | 267.45 | 281.55 | 266.70 | 264.20 | 7.5 | 27.49 | 1.94 |
| LDW | 45.27 | 52.34 | 54.91 | 52.38 | 51.37 | 0.985 | 31.08 | 1.88 |
| SPAD | 61.60 | 63.24 | 62.10 | 58.42 | 60.38 | 1.565 | 6.72 | 2.46 |
| FD cm | 4.56 | 4.79 | 5.81 | 5.96 | 5.29 | 0.0815 | 198.64 | 1.89 |
| FL cm | 16.81 | 17.16 | 17.91 | 19.60 | 17.55 | 0.68 | 16.05 | 3.9 |
| NF | 14.44 | 19.56 | 22.38 | 22.90 | 19.90 | 1.3 | 34.59 | 4.52 |
| AFW | 288.95 | 327.45 | 383.65 | 385.75 | 306.65 | 14.785 | 27.06 | 3.47 |
| TFY | 4.18 | 6.40 | 8.60 | 8.84 | 6.12 | 6.745 | 71.3 | 5.75 |
| EC | 0.53 | 0.70 | 1.01 | 1.25 | 1.21 | 0.23 | 9.62 | 13.5 |
| pH | 8.06 | 8.36 | 8.16 | 8.11 | 8.24 | 0.745 | 2.11 | 4.96 |
| CaCo3 | 7.27 | 7.14 | 6.78 | 6.47 | 7.90 | 0.48 | 7.31 | 4.63 |
| OM | 0.56 | 0.72 | 0.78 | 0.73 | 0.56 | 0.14 | 10.24 | 9.49 |

**Table S4. Treatment superiority based on LSD**

| Trait | Plant Height | | | Quality Traits | | | Vegetative Growth | |
| --- | --- | --- | --- | --- | --- | --- | --- | --- |
|  | **PH16** | **PH32** | **PH60** | **DM%** | **TSS** | **Anthocyanin** | **NB** | **NL** |
| Best treatment group and Ranking | T2(a)> T0, T1, T3, T4 (b) | T2(a) > T3(b) > T0,T1(c) > T4(bc) | T2(a) >T1(b) > T3 (b)>T4(b) > T0 (c) | T2(a) > T3(b) > T1(c) > T4(c) >  T0 (d) | T3(a) > T4(ab)> T2(ab)>  T1 (b)> T0 (c) | T2(a)>T3(b)> T4(c)>T1(d)> T0 (e) | T2(a)>T3(ab) >T1(bc)>T4(c) > T0 (d) | T2 (a) > T1 (b) > T3 (b) > T4 (c) > T0 (d) |
| Trait | **Biomass** | | **Yield Components** | | | **Yield** | | |
|  | **LFW** | **LDW** | **Fruit diameter** | **Fruit length** | **Fruit number** | **Average fruit weight** | **Total yield** | |
| Best treatment group and Ranking | T2 (a) > T1 (b) > T3 (b) > T4 (b) > T0 (c) | T2 (a) > T1 (b) T3 (b) >T4 (c) >T0 (d) | T3 (a) >T2 (b) > T4 (c) >T1 (d) > T0 (e) | T3 (a)> T2 (b)> T4 (c)> T1 (d)> T0 (e) | T3 (a)> T2 (a)> T4 (b)> T1 (c)> T0 (d) | T3(a), T2(a)> T1(b)>T4(b)> T0 (c) | T3(a)>T2(a) >T1(b) > T4(b) >T0(c) | |
